# Context-Dependent Roles for Toll-Like Receptors 2 and 9 in the Pathogenesis of Staphylococcus aureus Osteomyelitis

**DOI:** 10.1128/iai.00417-22

**Published:** 2022-10-13

**Authors:** Jenna R. Petronglo, Nicole E. Putnam, Caleb A. Ford, Virginia Cruz-Victorio, Jacob M. Curry, Casey E. Butrico, Laura E. Fulbright, Joshua R. Johnson, Sun H. Peck, Sana R. Fatah, James E. Cassat

**Affiliations:** a Department of Pathology, Microbiology, and Immunology, Vanderbilt University Medical Center, Nashville, Tennessee, USA; b Department of Biomedical Engineering, Vanderbilt University, Nashville, Tennessee, USA; c Department of Pediatrics, Division of Pediatric Infectious Diseases, Vanderbilt University Medical Center, Nashville, Tennessee, USA; d Vanderbilt Center for Bone Biology, Vanderbilt University Medical Center, Nashville, Tennessee, USA; e Division of Clinical Pharmacology, Department of Medicine, Vanderbilt University Medical Center, Nashville, Tennessee, USA; f Vanderbilt Institute for Infection, Immunology, and Inflammation (VI4), Vanderbilt University Medical Center, Nashville, Tennessee, USA; University of California, Davis

**Keywords:** osteoimmunology, osteomyelitis, Toll-like receptors, osteoclasts, *Staphylococcus aureus*, inflammation, innate immunity

## Abstract

Staphylococcus aureus is the major causative agent of bacterial osteomyelitis, an invasive infection of bone. Inflammation generated by the immune response to S. aureus contributes to bone damage by altering bone homeostasis. Increases in the differentiation of monocyte lineage cells into bone-resorbing osteoclasts (osteoclastogenesis) promote bone loss in the setting of osteomyelitis. In this study, we sought to define the role of Toll-like receptor (TLR) signaling in the pathogenesis of S. aureus osteomyelitis. We hypothesized that S. aureus-sensing TLRs 2 and 9, both of which are known to alter osteoclastogenesis *in vitro*, promote pathological changes to bone, including increased osteoclast abundance, bone loss, and altered callus formation during osteomyelitis. Stimulation of osteoclast precursors with S. aureus supernatant increased osteoclastogenesis in a TLR2-dependent, but not a TLR9-dependent, manner. However, *in vivo* studies using a posttraumatic murine model of osteomyelitis revealed that TLR2-null mice experienced similar bone damage and increased osteoclastogenesis compared to wild type (WT) mice. Therefore, we tested the hypothesis that compensation between TLR2 and TLR9 contributes to osteomyelitis pathogenesis. We found that mice deficient in both TLR2 and TLR9 (*Tlr2/9^−/−^*) have decreased trabecular bone loss in response to infection compared to WT mice. However, osteoclastogenesis is comparable between WT and *Tlr2/9^−/−^* mice, suggesting that alternative mechanisms enhance osteoclastogenesis *in vivo* during osteomyelitis. Indeed, we discovered that osteoclast precursors intracellularly infected with S. aureus undergo significantly increased osteoclast formation, even in the absence of TLR2 and TLR9. These results suggest that TLR2 and TLR9 have context-dependent roles in the alteration of bone homeostasis during osteomyelitis.

## INTRODUCTION

The Gram-positive bacterium Staphylococcus aureus causes devastating infections in a variety of tissue sites, including the musculoskeletal system. S. aureus is the most common causative agent of infectious osteomyelitis, or the infection of bone (1, 2). A major pathological feature of osteomyelitis is dysregulated bone homeostasis and consequent bone damage (3–5). In healthy skeletal tissue, bone-building osteoblasts and bone-resorbing osteoclasts balance the deposition of new bone matrix with the resorption of old bone, thereby maintaining bone homeostasis (6, 7). The perturbation of bone homeostasis during osteomyelitis leads to bone loss and aberrant bone healing and also contributes to morbid complications, including pathological fractures (3, 4, 8). Moreover, vascular damage occurring in response to infection can interfere with antibiotic delivery, thereby promoting chronic infection (3, 5, 8). Thus, in addition to prolonged antibiotic therapy, patients suffering from osteomyelitis often require invasive surgical debridement to remove infected and necrotic bone (2–4, 8). To create new therapeutic strategies that reduce morbidity and improve the antibiotic treatment of osteomyelitis, it is crucial to define the mechanisms by which bacterial infections incite bone damage. Thus, the objective of this study is to define how innate immune signaling, specifically through Toll-like receptor (TLR) sensing of S. aureus, influences pathological bone remodeling during osteomyelitis.

In inflamed bone, osteoclasts are key mediators of bone loss or osteolysis (9). Increases in osteoclast differentiation and bone resorption are recognized mechanisms of pathological osteoclast activation in inflammatory disease (9–11). In physiologic conditions, osteoclastogenesis occurs when precursors of the monocytic lineage encounter the canonical differentiation factor, receptor activator of the nuclear factor (NF)-κB ligand (RANKL) (12). RANKL-primed osteoclast progenitors then fuse to form large, multinucleated cells that are capable of resorbing bone. However, osteoclastogenesis is shaped by stimuli in the bone microenvironment, including the engagement of the pattern recognition receptors (PRRs) and the cytokine milieu (9, 13). Specifically, signaling through TLRs 2, 4, and 9 promotes osteoclastogenesis in RANKL-primed osteoclast precursors (13–15). Conversely, TLR signaling prior to RANKL can inhibit subsequent osteoclastogenesis (14, 16–18). Downstream of TLR signaling, proinflammatory cytokines such as IL-6, IL-1β, and TNF-α enhance osteoclast differentiation, while other cytokines, such as IL-10, are inhibitory (18–22). Thus, responses to bacterial infection have the potential to influence osteoclast formation directly through receptor ligation on progenitor cells, and indirectly by potentiating cytokine release from neighboring cells. Moreover, additional resident skeletal cells, including osteoblasts, also engage TLRs in response to S. aureus, which can perturb environmental RANKL and cytokine levels (23–25). In addition to bone loss, bone formation is also altered during osteomyelitis. In animal models of disease, exposure to live infection or staphylococcal pathogen-associated molecular patterns (PAMPs) promotes excessive periosteal callus formation. Rather than progressing to normal healing, these calluses can persist without effective mineralization and remodeling to leave mechanically weakened bone (26, 27). Impaired healing, abnormal callus deposition, and bone loss are also characteristic of human disease (26). Therefore, it is important to define how specific innate immune responses to bacterial pathogens disrupt bone homeostasis.

TLR2 and TLR9 have been reported to contribute to the innate immune response during S. aureus infection (28–31). TLR2 dimerizes with TLR1 and/or TLR6 to detect tri- and di-acylated bacterial lipoproteins, respectively, which activates NF-κB signaling (28, 29). TLR2 has been shown to promote host survival in septic infections, but its role is less clear in tissue-specific models of infection (32–38). TLR9 is localized to endosomal compartments and recognizes bacterial CpG-DNA (39). Studies on how TLR9 contributes to host defenses during S. aureus infections *in vivo* have been limited (31, 40–42). Moreover, many *in vitro* studies document the ability of TLR agonists to promote osteoclast differentiation and mature cell resorption, but the understanding of how these receptors impact osteoclastogenesis and bone homeostasis during bacterial infections *in vivo* is more limited. In this study, we investigate the role of TLR2 and TLR9 signaling in the specific context of posttraumatic S. aureus osteomyelitis with a focus on understanding how these immune receptors influence host antibacterial defenses and modulate infection-induced changes to bone homeostasis.

## RESULTS

### *S. aureus* supernatants promote osteoclast differentiation of RANKL-primed precursors in a TLR2-dependent manner.

**FIG 1:**
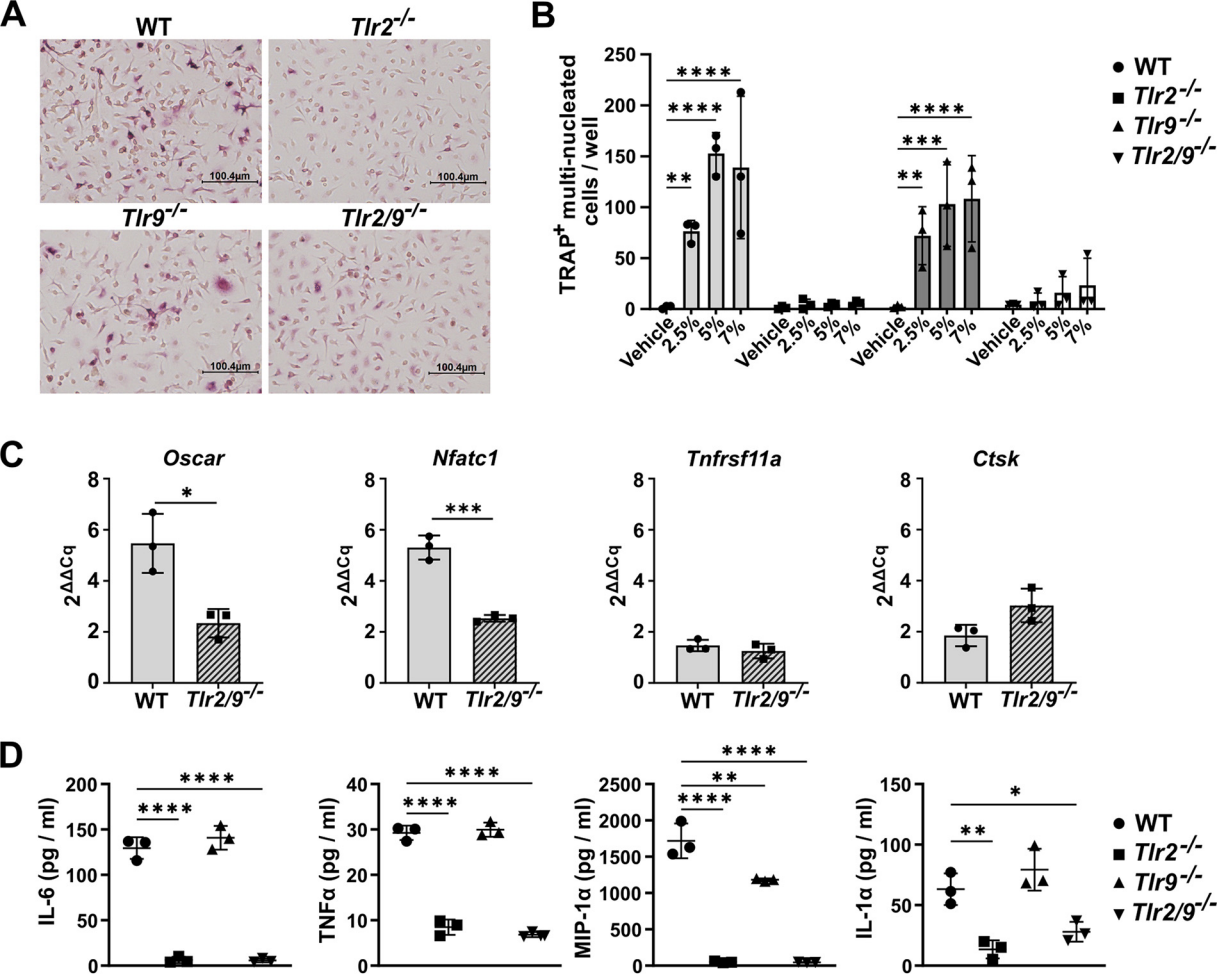
S. aureus supernatants promote osteoclastogenesis in RANKL-primed precursors through TLR2. (A and B) Bone marrow-derived monocytes (BMDMs) were isolated from wild type (WT), *Tlr2^−/−^*, *Tlr9^−/−^*, and *Tlr2/9^−/−^* mice and were cultured with 35 ng/mL RANKL + CMG 14-12 supernatant for 2 days. RANKL-primed osteoclast precursors were stimulated with the indicated vol/vol percentage of supernatant from the Δ*psmα1-4* strain of S. aureus or vehicle control in media containing CMG 14-12 supernatant but lacking RANKL. After 4 more days of culture, the cells were stained for tartrate-resistant acid phosphatase (TRAP), and TRAP^+^ multinucleated (≥3 nuclei) osteoclasts were quantified. (A) Representative images of the 5% treatment condition were obtained at 20× magnification. (B) Osteoclast counts were evaluated via two-way analyses of variance (ANOVA), and the counts from each genotype were compared to the vehicle via Dunnett’s multiple-comparisons test. **, *P* < 0.01; ***, *P* < 0.001; ****, *P* < 0.0001. If not denoted with asterisks, the differences between treatments were not statistically significant. Error bars denote the standard deviation (SD). The results are representative of three biological replicate experiments with *n* = 3 technical replicates plotted. (C) BMDMs were primed with 35 ng/mL RANKL and CMG 14-12 supernatant for 2 days and then stimulated with 7.5% supernatant from Δ*psmα1-4* or with vehicle for 24 h in media without RANKL. Cell lysates were collected, and transcript levels were measured using RT-qPCR. ΔΔCq values were compared between genotypes using *t* tests. *, *P* < 0.05; ***, *P* < 0.001. If not denoted with asterisks, the differences between genotypes were not statistically significant. Error bars denote the SD. The results are representative of two biological replicates with *n* = 3 technical replicates per group. (D) BMDMs were cultured in 35 ng/mL RANKL + CMG 14-12 supernatant for 2 days. RANKL-primed osteoclast precursors were stimulated with 7% Δ*psmα1-4* Δ*spa* supernatant in the absence of RANKL. The cell culture supernatants were collected after 12 h of stimulation. Cytokine abundance was measured using Luminex technology. Cytokines were compared across genotypes using one-way ANOVAs, and Dunnett’s multiple-comparisons test was used to compare between WT and knockout genotypes. *, *P* < 0.05; **, *P* < 0.01; ****, *P* < 0.0001. If not denoted with asterisks, the differences between genotypes were not statistically significant. Error bars denote the standard deviation (SD). Results represent one biological replicate with *n* = 3 technical replicates per group.

To explore the potential TLR-dependent effects of bacterial infection on bone homeostasis, we began by testing the effect of S. aureus supernatant stimulation on the differentiation of RANKL-primed bone marrow-derived monocytes (BMDMs), or osteoclast precursors, *in vitro*. We used supernatants prepared from the S. aureus LAC (AH1263) Δ*psmα1-4* strain in these experiments. We chose this strain for our model because we have observed that concentrated supernatants containing the alpha-type phenol soluble modulins (PSMα) are potently cytotoxic to skeletal cells (43, 44). We tested the extent to which Δ*psmα1-4* supernatant increased osteoclastogenesis in RANKL-primed precursors isolated from WT, *Tlr2^−/−^*, *Tlr9^−/−^*, and *Tlr2/9^−/−^* mice. We found that WT and *Tlr9^−/−^* RANKL-primed osteoclast precursors underwent significant osteoclastogenesis in response to S. aureus supernatant, compared to a vehicle control (Fig. 1A and B). Conversely, no significant differences were detected between supernatant- and vehicle-treated cells from *Tlr2^−/−^* and *Tlr2/9^−/−^* mice (Fig. 1B), implicating TLR2 as a major driver of osteoclastogenesis in RANKL-primed osteoclast precursors exposed to Δ*psmα1-4* supernatant.

To corroborate the results from the osteoclast enumeration, we measured the transcripts of the major osteoclast transcription factor *Nfatc1* (NFATc1) as well as three genes associated with osteoclastogenesis: *Oscar* (OSCAR), *Ctsk* (CathepsinK), and *Tnfrsf11a* (RANK receptor) (45–48). Transcriptional changes were measured in WT and *Tlr2/9^−/−^* RANKL-primed osteoclast precursors exposed to Δ*psmα1-4* supernatant, relative to vehicle. WT cells had a greater induction of *Oscar* and *Nfatc1* upon supernatant exposure compared to *Tlr2/9^−/−^* cells. The induction of *Ctsk* and *Tnfrsf11a* did not differ between genotypes (Fig. 1C). Because osteoclast differentiation can be augmented by osteoclastogenic cytokines, we next measured cytokine production in RANKL-primed cells responding to Δ*psmα1-4* supernatant. Bacterial supernatants were prepared from a double knockout Δ*psmα1-4* and staphylococcal protein A (Δ*spa*) strain because protein A can bind to the Fc regions of antibodies to interfere with antibody-based cytokine detection (49, 50). Release of osteoclastogenic cytokines, including IL-6, TNF-α, MIP-1α, and IL-1α, occurred in WT and *Tlr9^−/−^* cells upon stimulation with the bacterial supernatant. These responses were significantly reduced in cells lacking TLR2 (Fig. 1D; Table S1). Taken together, these results indicate that molecular changes consistent with osteoclastogenesis occur in RANKL-primed osteoclast precursors exposed to S. aureus supernatant, and these changes are blunted when TLR2 is absent.

### TLR2 promotes osteoclastogenesis during dual stimulation with bacterial supernatant and RANKL.

**FIG 2:**
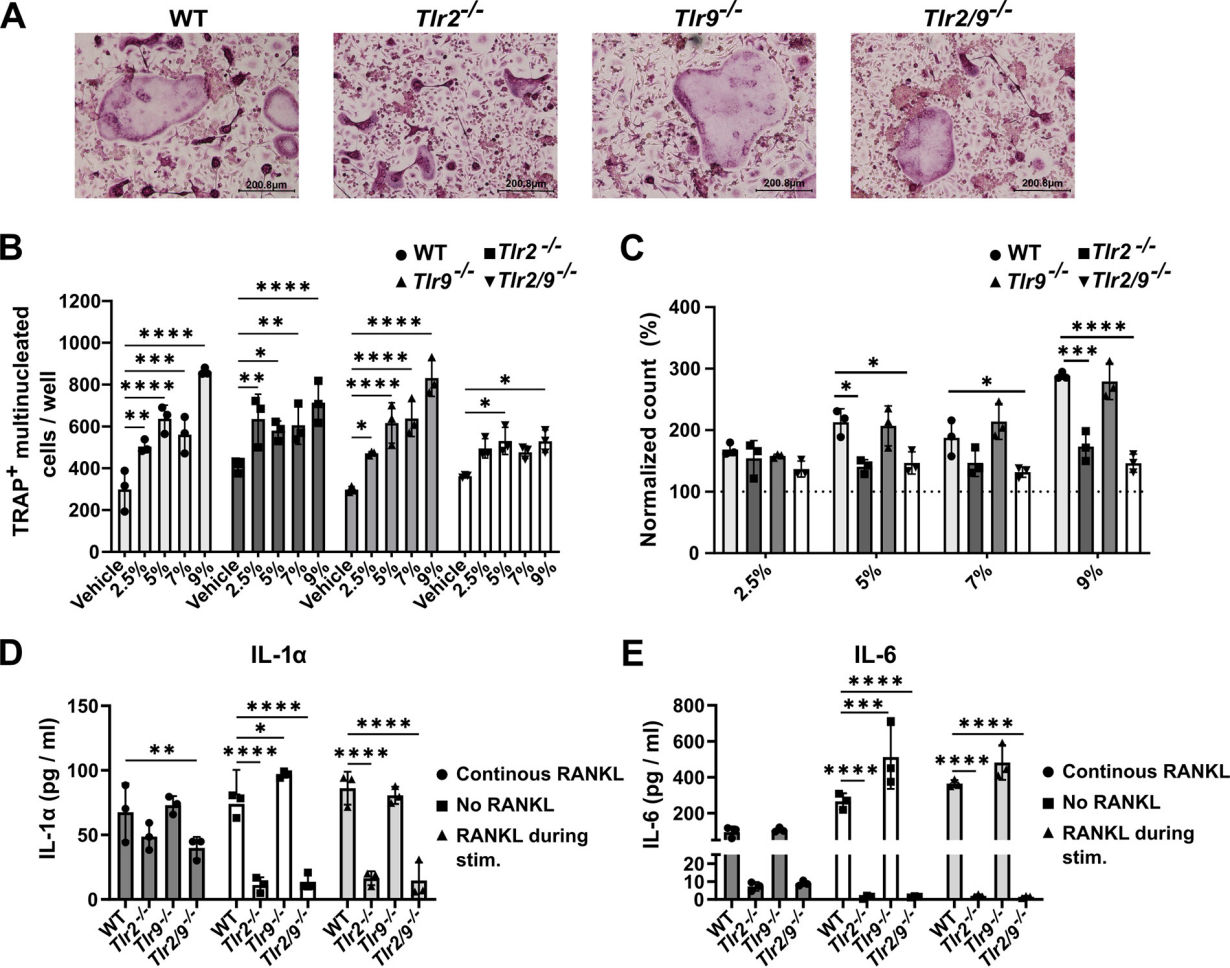
The timing of RANKL exposure alters the relative impact of TLR2 on bacterial-induced osteoclastogenesis and cytokine responses. (A–C) BMDMs from WT, *Tlr2^−/−^*, *Tlr9^−/−^*, and *Tlr2/9^−/−^* mice were plated in CMG 14-12 supernatant + 35 ng/mL RANKL. After 1 day of culture, RANKL and CMG 14-12 supernatant stimulation was maintained, and cells were exposed to the indicated vol/vol percentage of S. aureus supernatant from the Δ*psmα1-4* strain or the vehicle control. After 4 more days of culture, the cells were fixed and stained for TRAP, and TRAP^+^, multinucleated osteoclasts were quantified. (A) Representative images of the 5% treatment condition were taken at 10× magnification. (B) Counts of TRAP^+^ multinucleated cells were analyzed by a two-way ANOVA, and Dunnett’s multiple-comparisons test was used to compare between the treatment and the vehicle for each genotype. *, *P* < 0.05; **, *P* < 0.01; ***, *P* = 0.0001; ****, *P* < 0.0001. If not denoted with asterisks, the differences between treatments were not statistically significant. Error bars denote the SD. The results are representative of three biological replicates containing *n* = 3 technical replicates per group. (C) Changes in osteoclast number were calculated relative to the average vehicle count for the respective genotype. For each treatment, a one-way ANOVA was used to assess the effect of the genotype, and Dunnett’s multiple-comparisons test was used to compare each genotype to WT within each supernatant concentration. *, *P* < 0.05; **, *P* < 0.01; ***, *P* < 0.001; ****, *P* < 0.0001. If not denoted with asterisks, the differences between genotypes were not statistically significant. Error bars denote the standard deviation (SD). The results are representative of three biological replicates containing *n* = 3 technical replicates per group. (D and E) BMDMs were seeded in media with or without RANKL. After 2 days of culture, cells incubated in media without RANKL were treated with 7% vol/vol Δ*psmα1-4* Δ*spa* supernatant (“No RANKL”) or RANKL + 7% vol/vol Δ*psmα1-4* supernatant (“RANKL during stim.”). Cells incubated in media with RANKL were treated with 7% Δ*psmα1-4* Δ*spa* supernatant with RANKL maintained (“continuous RANKL”). After an additional 12 h of incubation, cytokines were measured from the cell culture media using Luminex technology. Cytokine concentrations were evaluated via two-way ANOVAs, and Dunnett’s multiple-comparisons test was used to compare each genotype to the WT. *, *P* < 0.05; **, *P* < 0.01; ***, *P* = 0.0001; ****, *P* < 0.0001. If not denoted with asterisks, the differences between genotypes were not statistically significant. Error bars denote the standard deviation (SD). The results represent one biological replicate with *n* = 3 technical replicates per group.

The fate of an osteoclast precursor is influenced by the agonism of TLR2 and 9, especially before or shortly after RANKL exposure (13, 14). However, in the bone microenvironment, exposure to RANKL and bacterial ligands may occur either sequentially or simultaneously. Thus, we questioned whether the ability of TLR2 to promote osteoclastogenesis would be altered when RANKL stimulation continued during S. aureus supernatant exposure. We measured osteoclast formation in WT, *Tlr2^−/−^*, *Tlr9^−/−^*, and *Tlr2/9^−/−^* BMDMs primed with RANKL for 1 day, followed by dual RANKL and Δ*psmα1-4* supernatant stimulation. We found that osteoclast abundance increased in response to a range of supernatant doses in WT, *Tlr2^−/−^*, and *Tlr9^−/−^* cells. However, *Tlr2/9^−/−^* cells incurred increased osteoclast formation only with select supernatant concentrations (Fig. 2A and B). To better visualize the increase in osteoclast formation incited by supernatant treatment and to control for stochastic differences in the baseline osteoclast number, the data were also analyzed after the normalization of osteoclast counts to the vehicle control for each genotype. This analysis revealed that the loss of TLR2, but not of TLR9, limits the increase in osteoclast differentiation, as *Tlr2^−/−^* and *Tlr2/9^−/−^* cells had significantly less induction of osteoclast formation than did WT cells following treatment with 5%, 7%, or 9% supernatant (Fig. 2C). These results suggest that TLR2 promotes increased osteoclastogenesis when RANKL and bacterial stimulation are applied concurrently. Because some increases in osteoclastogenesis occurred in *Tlr2^−/−^* cells, there are likely TLR2-independent mechanisms that are responsible for a proportion of the osteoclast formation that occurs in response to concurrent RANKL and bacterial stimulation.

### TLR2-dependent cytokine responses to bacterial supernatants are affected by the timing of RANKL exposure.

To better understand the TLR2-dependent signaling events that lead to the enhancement of osteoclastogenesis in the presence of RANKL, we measured cytokine responses in WT, *Tlr2^−/−^*, *Tlr9^−/−^*, and *Tlr2/9^−/−^* BMDMs exposed to three different sequences of RANKL and Δ*psmα1-4* Δ*spa* supernatant: (i) RANKL followed by Δ*psmα1-4* Δ*spa* supernatant with continued RANKL; (ii) no RANKL, followed by Δ*psmα1-4* Δ*spa* supernatant; or (iii) no RANKL, followed by RANKL and Δ*psmα1-4* Δ*spa* supernatant. We found that the presence of TLR2 was a strong determinant of the cytokine response, irrespective of RANKL exposure (Tables S2-4). However, the magnitude of the cytokine release was reduced by continuous RANKL exposure for most of the cytokines measured (Table S3). The release of the osteoclastogenic cytokines IL-6 and IL-1α was significantly affected by both cell genotype and by RANKL exposure when evaluated via a two-way analysis of variance (ANOVA). Moreover, both cytokines were reduced in *Tlr2^−/−^* and *Tlr2/9^−/−^* cells compared to WT cells when the cells were either not exposed to RANKL or simultaneously exposed to RANKL and supernatant, but not when the RANKL exposure was continuous (Fig. 2D and E). These data indicate that cytokine elaboration in response to S. aureus supernatants is influenced by prior RANKL exposure and occurs in a TLR2-dependent manner.

### Loss of TLR2 does not affect bacterial burdens during *S. aureus* osteomyelitis.

**FIG 3:**
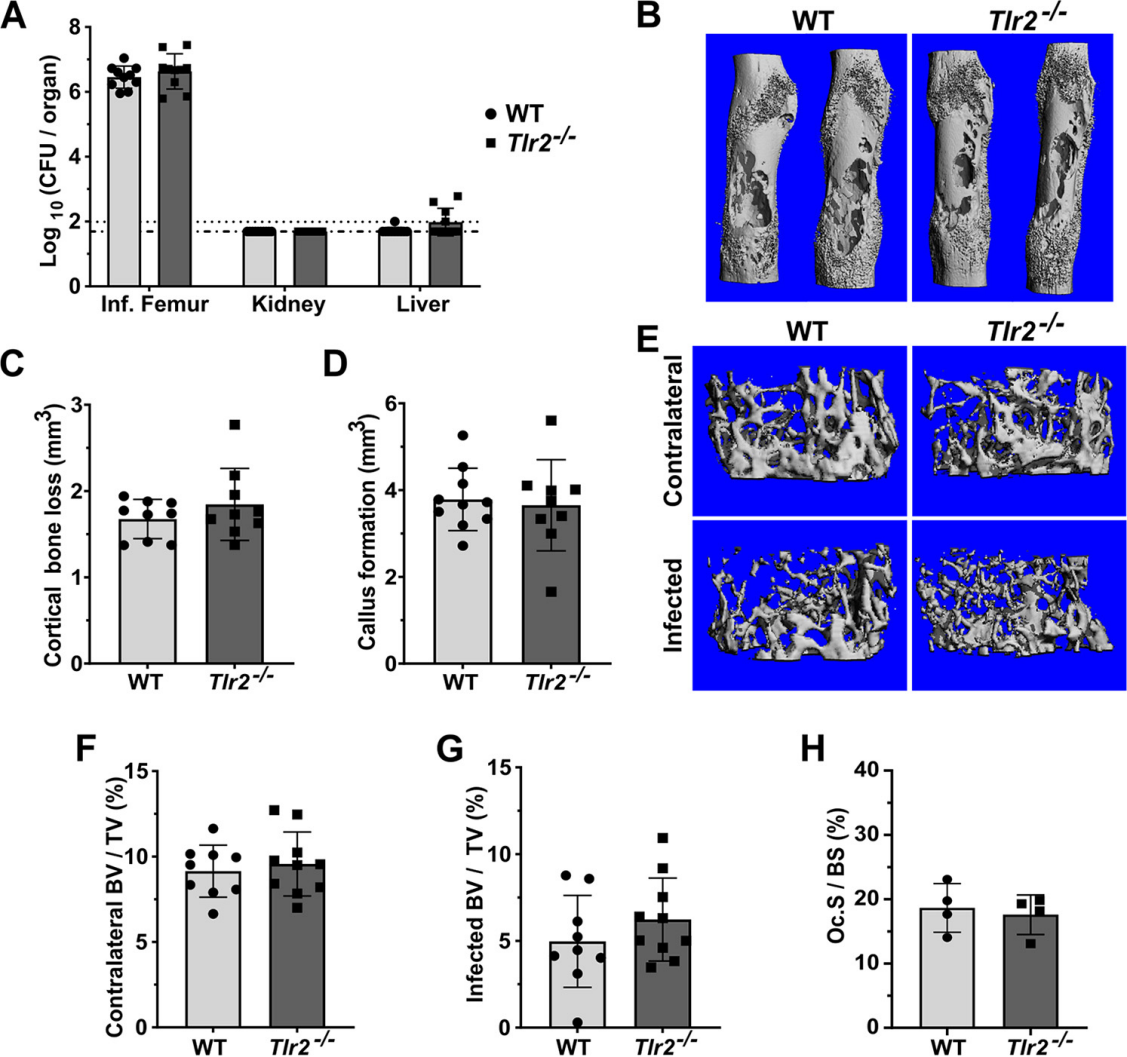
TLR2 is dispensable for the control of bacterial burdens and does not contribute to bone damage during S. aureus osteomyelitis. (A–H) Mice were subjected to osteomyelitis via intraosseous injection of 10^6^ CFU of S. aureus. Femurs were extracted on day 14 postinfection. For all graphs, error bars denote the SD. For all graphs, *, *P* < 0.05; **, *P* < 0.01; ***, *P* < 0.001; ****, *P* < 0.0001. If not denoted with asterisks, the differences between genotypes were not statistically significant. (A) Femurs, kidneys, and livers were extracted and homogenized for CFU enumeration. Dotted lines indicate the Log_10_ transformed limits of detection. The Log_10_ transformed CFU/femur values were compared between genotypes by unpaired *t* tests. Data were pooled from two independent experiments, *n* = 10 per genotype. (B–H) Infected and contralateral femurs were isolated. Bone parameters were assessed using microcomputed tomography (μCT). Results are compiled from two independent experiments. For cortical bone, *n* = 10 for WT and *n* = 9 for *Tlr2^−/−^*. For trabecular bone, *n* = 9 for WT and *n* = 10 for *Tlr2^−/−^*. (B) Representative 3D images of infected femurs were constructed by μCT. (C) Cortical bone loss was calculated using μCT, and were compared between genotypes using an unpaired *t* test. (D) Callus formation was measured by μCT, and compared between genotypes using an unpaired *t* test. (E) Representative images of trabecular bone in infected and contralateral femurs were constructed by uCT. Images represent the median infected femur % bone volume/total volume (%-BV/TV). (F) The %-BV/TV of contralateral femurs was calculated using μCT, and the values were compared using an unpaired *t*-test. (G) The %-BV/TV values of infected femurs were compared between genotypes using an unpaired *t*-test. (H) Histomorphometry was performed on TRAP-stained femur sections to measure TRAP^+^ cell surface on the trabecular bone, relative to total trabecular volume. The %-osteoclast surface/bone surface (Oc.S/BS) was compared between genotypes using a Mann-Whitney U test.

Because TLR2 has been shown to promote the host response to S. aureus, we hypothesized that the loss of TLR2 would alter the pathogenesis of osteomyelitis (32, 35, 36). We first tested whether the loss of TLR2 would compromise host containment of S. aureus during osteomyelitis. On day 14 postinfection (dpi), no significant differences in colony forming units (CFU) were detected in infected femurs from *Tlr2*^−/−^ mice compared to those of the WT mice. To determine whether enhanced dissemination from the inoculation site in the femur was occurring, we also measured the bacterial burdens in the liver and kidneys and found no significant differences between the genotypes (Fig. 3A). It has been reported that TLR2 signaling plays a role in host bacterial burden control at early time points during S. aureus skin infection (33, 51). To address the possibility that the loss of TLR2 causes deficits in bacterial burden control early during osteomyelitis, we performed a short time course using a reduced inoculum to increase the resolution on small differences in the bacterial load. At 1 dpi, there was a significant increase in femur CFU in the WT mice compared to the *Tlr2^−/−^* mice, but this difference was not detected at 2 or 5 dpi (Fig. S1A). These results indicate that TLR2 is not a major contributor to the control of bacterial burdens in this model of S. aureus osteomyelitis.

### Loss of TLR2 does not impact osteolysis or callus formation during *S. aureus* osteomyelitis.

Based on the observation that TLR2 is a major driver of S. aureus supernatant-stimulated osteoclastogenesis *in vitro*, we hypothesized that TLR2 contributes to pathological changes in cortical and trabecular bone during osteomyelitis. To test this hypothesis, we used microcomputed tomography (μCT) to measure and compare callus formation, cortical bone destruction, and trabecular bone volume in WT versus *Tlr2^−/−^* mice subjected to osteomyelitis. There were no significant differences in cortical bone loss, callus formation, or percent-trabecular bone volume/total volume (%-BV/TV) between genotypes (Fig. 3B to G). We also performed histomorphometry on histologic sections from infected femurs to evaluate osteoclastogenesis *in vivo* (52). We found no significant differences between genotypes (Fig. 3H). In summary, our data show that TLR2 does not significantly contribute to bone damage during post-traumatic osteomyelitis.

### The dual loss of TLR2 and TLR9 does not compromise bacterial burden control during osteomyelitis.

**FIG 4:**
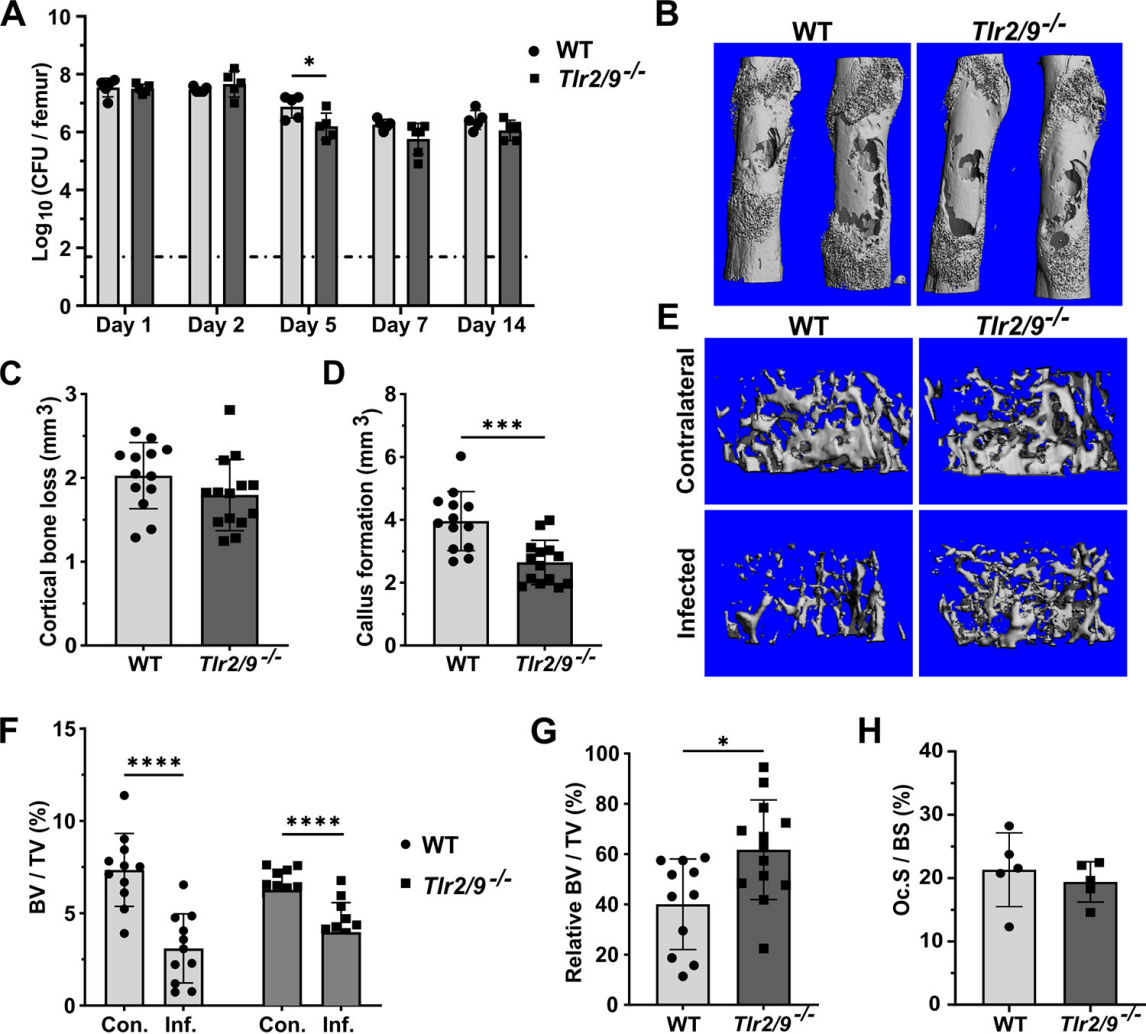
Combined TLR2- and TLR9-deficiency limits infection-induced trabecular bone loss and callus formation. (A–H) WT and *Tlr2/9^−/−^* mice were subjected to osteomyelitis via intraosseous injection of 10^6^ CFU of S. aureus. For all graphs, error bars denote the SD. (A) Femurs were collected for CFU enumeration on the indicated days postinfection. The dotted line indicates the Log_10_ transformed limit of detection. The Log_10_ CFU burdens were compared between genotypes using unpaired *t* tests (days 2, 5, and 14) or Mann-Whitney U tests (days 1 and 7), depending on data normality. For days 1, 2, 5, and 14, *n* = 5 per genotype, and *n* = 6 for day 7. *, *P* < 0.05. (B–H) Mice were euthanized on day 14 postinfection, and the infected and contralateral femurs were isolated. Bone parameters were assessed using μCT. The results are pooled from independent experimental replicates. For cortical bone, *n* = 13 for the WT, and *n* = 14 for the *Tlr2/9^−/−^* mice. For trabecular bone, *n* = 11 for the WT mice, and *n* = 13 for the *Tlr2/9^−/−^* mice. If not denoted with asterisks, the differences between genotypes were not statistically significant. (B) Representative 3D images of infected femurs were constructed using μCT. (C) Cortical bone loss was compared between genotypes using an unpaired *t* test. (D) Callus formation was compared between genotypes using an unpaired *t* test. (E) Representative images of trabecular bone in infected and contralateral femurs were constructed by uCT. Images represent the median infected femur %-BV/TV. (F) %-BV/TV values were analyzed by a repeated measures two-way ANOVA. ****, *P* < 0.0001 for limb type, *P* = 0.08729 for genotype, and ***, *P* = 0.004 for genotype × limb type. Sidak’s test was used to compare the %-BV/TV of the infected and contralateral limbs within genotypes. ****, *P* < 0.0001. (G) The infected femur BV/TV was normalized to the contralateral BV/TV, and the genotypes were compared using an unpaired *t* test, *, *P* = 0.0109. (H) Histomorphometry was performed on TRAP-stained femur sections to measure TRAP^+^ cell surface on trabecular bone, relative to total trabecular volume. %-osteoclast surface/bone surface (Oc.S/BS) was compared between genotypes using a Mann-Whitney U test.

Since we did not observe TLR2-dependent changes in bone pathology during S. aureus osteomyelitis, we hypothesized that other TLRs that are known to sense staphylococcal PAMPs compensate for the loss of TLR2 *in vivo*. While our *in vitro* studies did not indicate TLR9 to be a driver of osteoclastogenesis during exposure to bacterial supernatants, TLR9 agonism does modulate osteoclast differentiation *in vitro* in models using chemical agonism (13, 14, 17). Moreover, TLR9 is a major sensor of bacterial DNA and is suggested to modulate immune cell anti-staphylococcal activity *in vitro* (25, 31). Therefore, we next sought to determine the impact of the dual loss of TLR2 and TLR9 on the pathogenesis of S. aureus osteomyelitis. We first assessed whether *Tlr9^−/−^* mice had any defects in their ability to control bacterial burdens during osteomyelitis. No significant differences in femur, kidney, or liver bacterial burdens were detected between WT and *Tlr9^−/−^* mice at the end of infection on day 14 (Fig. S1B). Next, we induced S. aureus osteomyelitis in *Tlr2/9^−/−^* mice. At 1 and 2 dpi, there were no differences in the femur bacterial burdens between *Tlr2/9^−/−^* and WT mice. At 5 dpi, there was a modest but significant decrease in the bacterial burdens of the infected femurs of *Tlr2/9^−/−^* mice, but this difference was no longer evident by 7 dpi, and there were no significant differences at 14 dpi (Fig. 4A). To assess whether the loss of TLR2 and TLR9 affects host control of bacterial dissemination to organs outside the infected femur, we measured CFU burdens in the contralateral femur, kidneys, and liver over a 14-day time course of infection. No significant differences were detected on 1, 2, 5, 7, or 14 dpi (Fig. S2A–C). Moreover, the experiments were repeated using a lower inoculum dose to ensure that differences in bacterial burdens between genotypes were not obscured by high bacterial loads. No significant differences were detected at the midpoint of infection on 7 dpi (Fig. S2D).

### The dual loss of TLR2 and TLR9 alters callus formation and reduces trabecular bone loss during osteomyelitis.

To determine whether the loss of both TLR2 and TLR9 alters bone homeostasis during infection, we performed μCT on infected femurs from WT and *Tlr2/9^−/−^* mice collected at 14 dpi. There were no differences in cortical bone loss between WT and *Tlr2/9^−/−^* mice (Fig. 4B and C). However, *Tlr2/9^−/−^* mice had a significant decrease in callus formation compared to WT mice (Fig. 4D). To determine whether the loss of TLR2 and TLR9 alters infection-associated trabecular bone loss, we first performed baseline analyses of uninfected mice to rule out underlying differences in trabecular bone volumes within groups. There were no differences in trabecular measurements between uninfected *Tlr2/9^−/−^* and WT mice (Fig. S3A–D). In the infected mice, contralateral and infected femur %-BV/TVs from WT and *Tlr2/9^−/−^* femurs were compared using a two-way ANOVA. This analysis revealed that a significant amount of the variation in these measurements came from the limb type (contralateral versus infected) and from an interaction between the limb type and the mouse genotype (Fig. 4F). *Post hoc* analyses revealed significant differences between the contralateral and infected %BV/TVs for both *Tlr2/9^−/−^* and WT mice. To better understand the interaction between bone volume and TLR2/9-deficiency, we normalized the %BV/TV value of the infected femur to that of the contralateral femur for each mouse. This analysis revealed that the infected *Tlr2/9^−/−^* mice had significantly greater relative trabecular bone volumes compared to WT mice (Fig. 4G). To test whether the loss of TLR2 and TLR9 reduces infection-associated osteoclastogenesis, we assessed osteoclast abundance relative to bone volume in the trabecular region using histomorphometry (52). We found no significant differences in osteoclast surface per bone surface between genotypes (Fig. 4H). Taken together, these observations suggest that the dual loss of TLR2 and TLR9 significantly reduces reactive callus formation during infection. TLR2/9-deficiency also reduces relative trabecular bone loss, albeit modestly and without a significant reduction in relative osteoclast surface.

### TLR2- and TLR9-independent mechanisms promote osteoclastogenesis in RANKL-primed precursors during intracellular bacterial infection.

**FIG 5:**
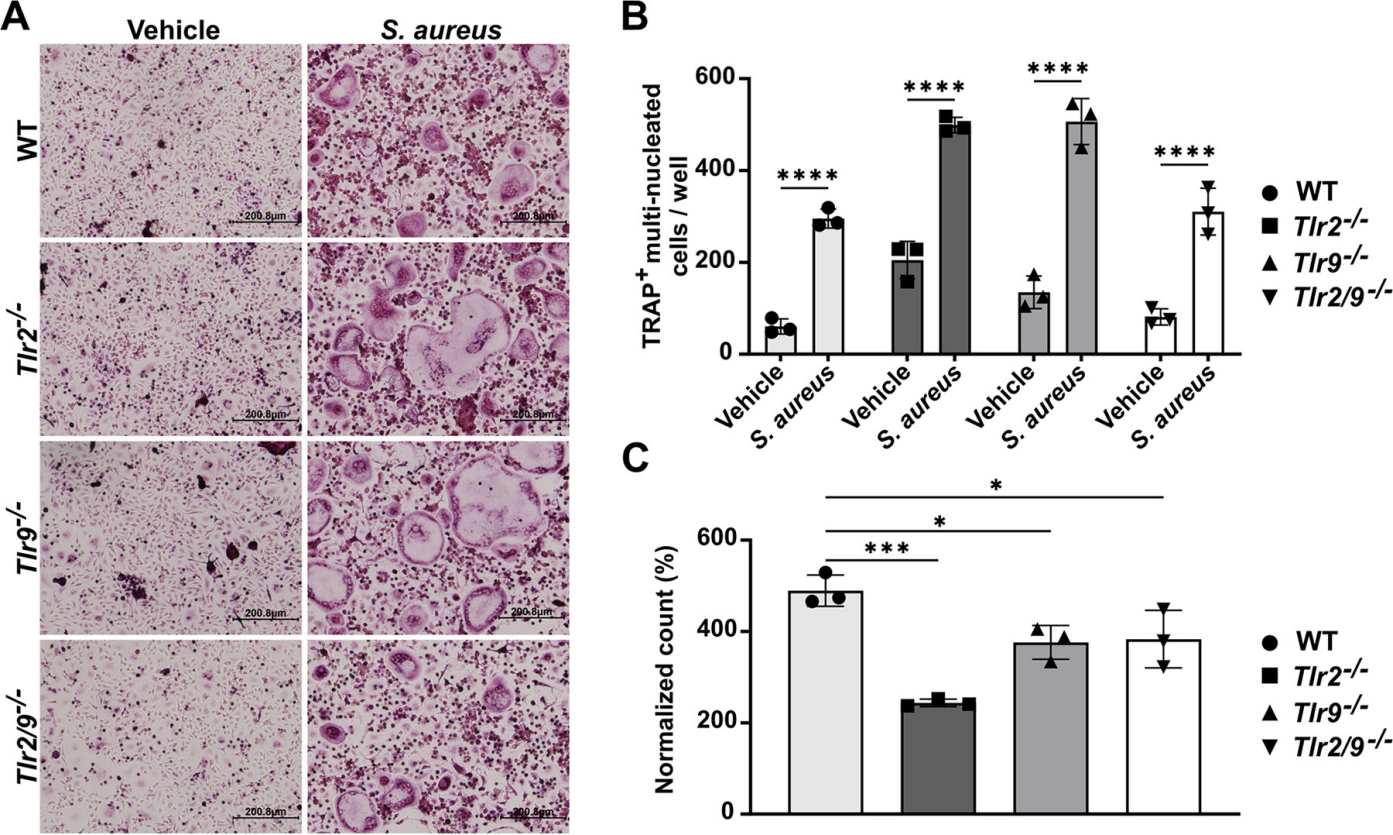
Increased osteoclastogenesis in RANKL-primed precursors infected with S. aureus is only partially mediated by TLR2 and TLR9. (A–C) BMDMs were cultured with 35 ng/mL RANKL + CMG 14-12 supernatant for 2 days. The cells were then infected with S. aureus at an MOI of 25 in the absence of RANKL, using gentamicin protection. For all, *, *P* < 0.05; **, *P* < 0.01; ***, *P* < 0.001; ****, *P* < 0.0001. If not denoted with asterisks, the differences between genotypes were not statistically significant. Error bars denote the SD, with *n* = 3 technical replicates per group. The data are representative of 3 independent experiments. (A) Representative images of TRAP-stained cells at day 2 postinfection. (B) After 2 days of infection, the cells were fixed and stained, and TRAP^+^ multinucleated (3 ≥ nuclei) osteoclasts were quantified. A two-way ANOVA was used to assess the effects of genotype and treatment, and Sidak’s test was used to compare each vehicle to S. aureus for each genotype. (C) The counts were normalized to the vehicle and were analyzed using a one-way ANOVA. Dunnett’s multiple-comparisons test was used to compare each genotype to the WT.

Our *in vivo* findings suggest that TLR2 and TLR9 modestly impact infection-induced bone loss and do not alter osteoclastogenesis in trabecular bone *in vivo*. To identify whether S. aureus can promote osteoclastogenesis through TLR2- or TLR9-indepenent mechanisms, we next studied the response of RANKL-primed osteoclast precursors to intracellular infection with WT S. aureus. We hypothesized that osteoclast precursors deficient in TLR2 and/or TLR9 would maintain the ability to differentiate through bacterial activation of alternative immune pathways. We infected RANKL-primed BMDMs from WT, *Tlr2^−/−^*, *Tlr9^−/−^*, and *Tlr2/9^−/−^* mice and found that, for all genotypes, infection significantly increased osteoclast numbers compared to those observed in the vehicle (Fig. 5A and B). To control for stochastic differences in osteoclast formation, the counts were also normalized to the vehicle control for each genotype to quantify the relative magnitude of increase in osteoclastogenesis resulting from infection. This analysis revealed that all genotypes had increased osteoclastogenesis from infection; however, WT cells incurred significantly greater infection-induced osteoclastogenesis than did cells lacking TLR2, TLR9, or TLR2/9. (Fig. 5C). To confirm that differences in the bacterial load did not contribute to differences in the osteoclast numbers identified between genotypes, CFU were enumerated from cell lysates immediately following infection and at 2 dpi. There were no significant differences in bacterial burdens between genotypes immediately after bacterial internalization or at 2 dpi (Fig. S4A–B). From these data, we conclude that TLR2 and TLR9 are partially responsible for infection-induced osteoclastogenesis in RANKL-primed cells, but TLR2- and TLR9-independent mechanisms contribute significantly to osteoclast formation during intracellular infection.

## DISCUSSION

Dysregulation of bone homeostasis is a hallmark of osteomyelitis pathogenesis in both animal models and human disease (2, 3, 27, 43). This manifests as both bone loss and abnormal bone formation. While the effects of S. aureus toxins contribute to the perturbation of bone homeostasis through direct cell lysis, previous studies indicate that inflammation generated by host bacterial sensing also contributes to altered bone homeostasis (27, 34, 43, 53–55). The presence of inflammatory cytokines in the bone microenvironment and direct PRR engagement can alter the differentiation and function of bone-resorbing osteoclasts, leading to bone loss (9, 44, 56, 57). Thus, defining the specific pathogen sensing receptors and immune signaling pathways that contribute most to the dysregulation of bone homeostasis is a vital prerequisite to understanding how to limit bone damage during osteomyelitis. In this study, we sought to determine how two PRRs capable of sensing staphylococcal PAMPs, TLR2 and TLR9, contribute to the control of the bacterial burdens and the dysregulation of bone homeostasis in the specific context of post-traumatic osteomyelitis.

*In vitro* experiments in this study focused on determining the consequences of S. aureus-mediated ligation of TLR2 and TLR9 in osteoclast lineage cells. Prior work has shown that the engagement of TLRs 2, 4, and 9 in RANKL-primed osteoclast precursors via purified agonists increases osteoclast formation (13, 14). Positive regulation of osteoclastogenesis through TLR engagement is associated with increases in c-Fos expression and with parallel changes to the cytokine milieu (14). Our results are in line with these observations, as S. aureus supernatant promoted osteoclastogenesis in RANKL-primed precursors in a TLR2-dependent manner. However, this finding did not translate *in vivo*, where TLR2-deficiency neither significantly altered the parameters of bone homeostasis nor affected the relative osteoclast surface. Thus, although the role of TLR2 in driving osteoclastogenesis has been relatively straightforward in cell culture studies, our *in vivo* results suggest that TLR2 plays a less crucial role in bone loss during osteomyelitis. This finding is reflected in previous publications that demonstrate that the role of TLR2 in bone loss varies by infection model (34, 55, 58, 59). The most unequivocal evidence that TLR2 can act as a modulator of trabecular bone loss comes from a 2013 study in which mice were intravenously administered formaldehyde-fixed S. aureus or chemical TLR2 agonists (58). Trabecular bone loss occurred in a TLR2-dependent manner, in line with parallel *in vitro* experiments (58). The disparity between these data and the current study may reflect the differences in modeling systemic TLR2 engagement with purified PAMPs versus infection with live bacteria, which are capable of dynamic metabolism, virulence factor production, and replication, which can provide a wide variety of pathogen-derived molecules for host detection.

The potential for compensation between innate immune signaling pathways provided a rationale for testing the effect of the dual loss of TLR2 and TLR9, with the expectation that *Tlr2/9^−/−^* mice would have reduced bone loss, reduced osteoclast surface, and less dysregulation of bone homeostasis during osteomyelitis. *Tlr2/9^−/−^* mice had a modest attenuation of trabecular bone loss, supporting our hypothesis that compensation between TLR2 and TLR9 limited the effects of TLR2-deficiency on bone loss. However, there was no concomitant decrease in relative osteoclast surface. Taken alongside data showing that TLR2- and TLR9-deficient osteoclast precursors undergo osteoclastogenesis upon intracellular infection with S. aureus, these data suggest that TLR2- and TLR9-independent pathways have the potential to modulate osteoclastogenesis during osteomyelitis. For instance, the detection of damage-associated molecular patterns (DAMPs) could be contributing to inflammatory bone damage through other innate immune receptors. TLR4 has been shown to promote osteoclast-mediated bone erosion during arthritis through the detection of neutrophil-derived S100A8/A9, presenting the possibility that TLR4 plays a role in osteomyelitis, even in those cases triggered by Gram-positive pathogens (60). Moreover, cell types outside of the osteoclast compartment may influence osteoclastogenesis in a cell-extrinsic manner that is not solely dependent on TLR2 and TLR9. Because prior exposure to RANKL determines whether a BMDM can differentiate into an osteoclast upon TLR engagement, it is possible that changes to osteoclastogenesis may occur in *Tlr2^−/−^* and/or *Tlr2/9^−/−^* mice, but, because of the opposing effects of TLR ligation pre- and post- RANKL, the net osteoclastogenesis remains unchanged (13, 14, 16, 17, 61). Exposure to RANKL blunted the release of most of the cytokines we measured. However, RANKL-primed WT and TLR2-null cells retained the ability to produce small amounts of IL-6 and IL-1α, presenting the possibility that, in microenvironments containing RANKL, NF-κB activation through the RANK receptor could augment cytokine release downstream of the TLRs. Thus, the *in vitro* and *in vivo* findings presented here support the notion that TLR2- and 9-independent mechanisms, including the engagement of other PRRs, cytokine signaling, and the activation of undiscovered innate immune pathways, function in osteoclast lineage cells to drive pathological changes to bone during S. aureus infection.

We observed that *Tlr2/9^−/−^* mice had significant decreases in callus formation. Although callus formation is a normal part of fracture repair, we observed that mice with osteomyelitis did not reach healing resolution around the bacterial inoculation site (43, 44, 62). Thus, excessive callus formation is aberrant in the setting of infection. Our results suggest that TLR2 and TLR9 influence bone forming cells, likely mesenchymal stromal cells such as osteoblasts, to promote callus formation. TLR signaling can occur in osteoblasts, with several articles reporting that TLR2 and TLR4 signaling alters osteoblast differentiation and RANKL presentation (24, 63–65). However, *in vivo*, the loss of TLRs 2, 4, and 9 did not alter fracture repair in a calvarial model of bone healing, while the abrogation of interleukin-1 type 1 receptor (IL-1R1) signaling significantly enhanced defect closure (57). Thus, it is possible that TLR2 and TLR9 alter osteoblast activity during osteomyelitis by modulating the elaboration of IL-1 cytokines.

Our results demonstrate that neither TLR2 nor TLR9 significantly contributes to the host control of bacterial burdens in our model of post-traumatic osteomyelitis, suggesting that other pathways may play more prominent roles in constraining S. aureus infections in bone. Evidence that TLR2 is critical to the host defense against S. aureus originated in a mouse model of intravenous septic infection, where receptor deficiency significantly accelerated host mortality (32). Furthermore, in a S. aureus craniotomy biofilm infection model, TLR2, but not TLR9, was critical for host bacterial containment through the caspase-1-mediated activation of IL-1β, which was reduced in the *Tlr2^−/−^* mice (35). Despite these findings, the conclusion that TLR2 exerts a significant effect on host anti-staphylococcal defenses has not been fully supported by all studies, with results varying based on tissue specificity and the infection model (32–36, 38, 55). Additionally, in sepsis, only mice deficient in myeloid differentiation factor 88 (MyD88) incurred significantly increased bacterial burdens in the blood and organs at multiple points, highlighting that MyD88-driven signaling pathways, in addition to TLR2, heavily contribute to anti-staphylococcal defenses (32). Cytokine elaboration likely underlies the MyD88-driven host control of bacterial burdens in disparate infection sites, even when different upstream receptors are required for activation. To this point, the activation of cytokines, namely, IL-1β and TNF-α, are critical for host control of bacterial expansion and immune cell recruitment in bone and implant-associated infection (35, 44, 66). Previous findings in our model of osteomyelitis corroborate the importance of IL-1R1 cytokines in bacterial containment during infection (44).

Our study has limitations that should be considered when interpreting the data. First, because we relied mainly on *in vitro* measurements of osteoclastogenesis to provide the rationale for our *in vivo* studies and to substantiate our *in vivo* findings, conclusions regarding TLR2- or TLR9-dependent changes to cellular function, such as osteoclast resorption, cannot be drawn from the data. Additionally, previous studies have indicated that cytokines are major drivers of osteoclast bone resorption during infection *in vitro* with S. aureus and can drive osteoclastogenesis *in vivo* (44, 67). To better understand why mice lacking TLR2 and TLR9 did not have significant decreases in osteoclastogenesis or changes to bacterial burden control *in vivo*, it will be important to understand how cytokines are affected by the loss of these receptors. IL-1R1 cytokines are major influencers of bone pathology during osteomyelitis and, more generally, in inflammatory bone loss (44, 57). Thus, exploration into the pathways upstream of inflammasome activation will be critical in future work. Finally, our studies revealed a strong impact of TLR2 and TLR9 on callus formation. Studying osteoblast-mediated bone pathology during osteomyelitis is also an important future direction for this work.

Overall, this study shows that, despite the clear role of TLR ligation in governing osteoclast differentiation in cell culture, TLR2 and TLR9 signaling contributes only modestly to inflammatory bone loss during S. aureus osteomyelitis. Importantly, the reduction in bone loss came without a significant change in the relative osteoclast surface in trabecular bone. Interestingly, our work shows that TLR2 and TLR9 may contribute to callus formation. Because the loss of TLR2 and TLR9 did not alter the control of bacterial burdens, other innate immune pathways may be more influential in promoting host responses to osteomyelitis. Continued work on the role of inflammation-generating host responses will be critical to uncovering the specific mechanisms at play in osteolysis during infection.

## MATERIALS AND METHODS

### Animal use.

C57BL/6J mice (Jackson stock number 000664) were used for all of the *in vivo* and *in vitro* experiments that employed wild type (WT) animals as controls. WT control mice used in the osteomyelitis model were ordered from The Jackson Laboratory (Bar Harbor, ME) and delivered to our housing facility 1 to 2 weeks prior to undergoing surgery. The *Tlr9^−/−^* mice were provided by Richard Peek (Vanderbilt University Medical Center) and bred in-house via homozygous crosses to generate experimental animals (39). The *Tlr2^−/−^* mice were ordered from Jackson (stock number 004650) and were either used for experiments following a 1 to 2-week equilibration period or were bred to maintain the knockout colony. To generate mice deficient in TLR2 and TLR9 (*Tlr2/9^−/−^* mice), the *Tlr2^−/−^* and *Tlr9^−/−^* mice were crossed to yield homozygous offspring. The offspring were genotyped using Transnetyx, Inc. (Cordova, TN). The *Tlr2/9^−/−^* colony was subsequently maintained via homozygous breeding. Because these strains were expected to be immunocompromised, the *Tlr2^−/−^*, *Tlr9^−/−^*, and *Tlr2/9^−/−^* mice were housed with sterile bedding and were provided sterile food.

### Bacterial strains and growth conditions.

Experiments were performed using either S. aureus USA300-lineage strain AH1263 (WT), a derivative strain lacking the alpha-type phenol-soluble modulins (Δ*psmα1-4*), or a derivative strain lacking the alpha-type phenol-soluble modulins and staphylococcal protein A (Δ*psmα1-4* Δ*spa*) (43, 68). Bacterial cultures used in both the *in vitro* and the *in vivo* infection experiments were grown overnight at 37°C in tryptic soy broth (TSB) with shaking at 180 rpm. 10 μg/mL erythromycin was added to the culture media for the Δ*psmα1-4* and Δ*psmα1-4* Δ*spa* strains. Bacterial inocula were prepared by back-diluting overnight cultures 1:100 into TSB and then incubating them for an additional 3 h at 37°C with shaking at 180 rpm. The subcultures were then centrifuged for 5 min at 4,000 × *g* to pellet the cells. The bacterial inoculum was adjusted to the desired concentration by dilution in 1× phosphate-buffered saline (PBS) and measuring the optical density. All postinfection CFU were enumerated after growth on trypticase soy agar (TSA) plates.

### Preparation of bacterial supernatants.

Concentrated bacterial supernatants were prepared as previously reported (43, 44). In brief,1 to 2 bacterial colonies were inoculated into 250 mL Erlenmeyer flasks containing 50 mL of sterile RPMI medium with 1% Casamino Acids (Fisher Scientific). Flasks were sealed with rubber stoppers and cultured for 15 h at 37°C with shaking at 180 rpm. Then, to pellet bacteria, cultures were centrifuged at 4,000 × *g* for 10 min. Supernatants were sterilized using a 0.22 μm polyethersulfone filter and then concentrated to a final volume of ~1.5 mL using an Amicon Ultra 3-kDa nominal molecular weight column (MilliporeSigma, Burlington, MA). The supernatants were again filter sterilized, and single-use aliquots were prepared on ice and frozen at −80°C.

### Whole bone marrow isolation and bone marrow-derived monocyte enrichment.

Whole bone marrow (WBM) was isolated from male WT, *Tlr2^−/−^*, *Tlr9^−/−^*, or *Tlr2/9^−/−^* mice aged between 8 and 12 weeks. WBM was flushed from femurs using unsupplemented MEM-α medium (Gibco, Waltham, MA), passed through a 100 μM filter, and centrifuged at 1,500 rpm × 5 min. Red blood cells were lysed in ammonium-chloride-potassium buffer for 10 min, and then 1× PBS was added to deactivate lysis. The cells were centrifuged again and resuspended in 100% fetal bovine serum (FBS) for counting. WBM cell preparations were then frozen in 10% dimethyl sulfoxide (DMSO) in FBS and stored in liquid nitrogen until use. Cryopreserved vials of WBM cells were rapidly thawed in a 37°C water bath and then washed in MEM-α medium supplemented with 10% FBS and 1× penicillin-streptomycin to remove the DMSO. To enrich for bone marrow-derived monocytes (BMDMs), WBM cells were seeded at 8 to 10 million cells per 10 cm tissue culture-treated dish in MEM-α medium + 10% FBS + 1× penicillin-streptomycin supplemented with macrophage colony-stimulating factor (M-CSF). Supernatant from the CMG 14-12 cell line served as the M-CSF source and was added at a vol/vol ratio of 1:5 to provide a high dose of M-CSF for the positive selection of monocytes (69). After 4 days of differentiation, BMDMs were scraped off 10 cm dishes and seeded into 96-well tissue culture plates at 50,000 cells/well or into 24-well plates at 250,000 cells/well. All cell cultures were incubated at 37°C with 5.0% CO_2_.

### Osteoclastogenesis assays.

Two osteoclastogenesis assays were used: a precommitment assay in which BMDMs were primed with RANKL for 48 h and then stimulated with bacterial supernatant in the absence of RANKL, and a continuous assay, in which RANKL stimulation was present during the duration of the bacterial stimulation. For each assay, the culture medium was as follows: MEM-α + 1× Penicillin-Streptomycin + 10% FBS with CMG 14-12 supernatant at 1:20 vol/vol (BMDM maintenance media) or MEM-α + 1× Penicillin-Streptomycin + 10% FBS with CMG 14-12 supernatant at 1:20 vol/vol and 35 ng/mL RANKL (osteoclastogenic media). In the precommitment osteoclastogenesis assay, BMDMs were cultured in osteoclastogenic media for 48 h. Then, the cells were washed once in warm 1× PBS, and the media were replaced with BMDM maintenance media. 2.5 to 7% vol/vol Δ*psmα1-4* supernatant or 7% vehicle control (1% Casamino Acids in RPMI) was added to the cells. 4 days later, the cells were fixed and stained for tartrate-resistant acid phosphatase (TRAP) and with 4′,6-diamidino-2-phenylindole (DAPI), as described below. In the continuous RANKL assay, BMDMs were plated in osteoclastogenic media. On the second day of culture, 2.5 to 9% vol/vol of concentrated supernatant prepared from the Δ*psmα1-4* strain or a 7% vehicle control (1% Casamino Acids in RPMI) was added to the cell monolayers. On the fourth day of culture, the medium was changed, and the supernatants/vehicle were replenished. On day 6 of culture, the cells were fixed and stained, as described below.

### TRAP and DAPI staining.

Adherent cells from the precommitment and continuous RANKL assays were fixed in a solution containing 4% formaldehyde and 0.05% Triton X-100 solution in 1× PBS for 10 min and then in a 1:1 acetone:ethanol solution for 1 min. The fixed cells were then TRAP-stained with reagents from the Acid Phosphatase, Leukocyte (TRAP) Kit (Sigma, number 378A). The TRAP-stained wells were also stained with DAPI to visualize the nuclei. Entire wells were imaged at 4× magnification using a Biotek Cytation 5 Imaging Reader (Agilent, Winooski, VT), and then the images were stitched together. The stitched color brightfield and DAPI images were merged and blinded prior to osteoclast quantification. Osteoclasts, defined as TRAP^+^ cells with 3 or more nuclei, were enumerated from images using Fiji ImageJ (70).

### Reverse transcriptase quantitative polymerase chain reaction (RT-qPCR).

BMDMs were cultured for 2 days in osteoclastogenic media. The cells were washed once with 1× PBS, and then BMDM maintenance medium was added to the wells. The cells were then stimulated with 5% vol/vol Δ*psmα1-4* supernatant or vehicle. After 24 h of stimulation, the cells were washed with 1× PBS and lysed using RT Lysis Buffer (Qiagen, Germantown, MD) supplemented with β-mercaptoethanol. Cell lysates were obtained via centrifugation in a Qiashredder column (Qiagen) at 13,000 rpm for 2 min. RNA was extracted from the cell lysates using the RNeasy Minikit (Qiagen) in accordance with the manufacturer’s instructions. The RNA concentrations were measured using a Biotek Synergy HT Microplate Reader (Agilent Winooski, VT). 500 ng of RNA per sample were used to synthesize cDNA using qScript XLT cDNA Supermix (QuantaBio, Beverly, MA). The cDNA was diluted 1:10 in nuclease-free water and frozen at −20°C until use. RT-qPCR was performed using iQ Sybr green Supermix (Bio-Rad, Hercules, CA) using the primer sequences shown (Table S5). The Cq values were analyzed using the ΔΔCq method (71).

### Multiplexed cytokine quantification.

BMDMs from WT, *Tlr2^−/−^*, *Tlr9^−/−^*, and *Tlr2/9^−/−^* male mice were plated at 50,000 cells/well in 96-well plates in either BMDM maintenance media or osteoclastogenic media. After 2 days of culture, the cells were washed in 1× PBS. The media were changed to either BMDM maintenance media or osteoclastogenic media, and then the cells were stimulated with 7% (vol/vol) Δ*psmα1-4 Δspa* supernatant so that the following culture conditions were achieved: RANKL exposure before supernatant treatment, RANKL exposure before and during supernatant treatment, RANKL exposure during supernatant treatment only, and no RANKL exposure before or during supernatant exposure. As a control, one group of WT cells in each RANKL exposure condition received vehicle treatment. After 12 h of stimulation, cell culture supernatants were collected and stored at −80°C until ready for analysis. For cytokine measurement, the samples were transitioned to −20°C overnight, thawed on ice the following day, and centrifuged at 2,000 × *g* for 10 min at 4°C to remove debris. Cytokine concentrations were measured from cell culture supernatants using the 25-plex Mouse Cytokine/Chemokine Magnetic Bead Panel (MilliporeSigma, Burlington, MA, catalog number MCYTOMAG-70K-PMX) on a FLEXMAP 3D instrument following the manufacturer’s instructions. Cytokine measurements below the limit of detection for a given analyte were reported as the lowest value on the standard curve. Values above the limit of detection were reported but not used in calculations or statistical comparisons.

### *In vitro* infection by gentamicin protection assay.

BMDMs were plated at a density of 50,000 cells/well in 96-well plates in osteoclastogenic media. After 2 days in culture, the cells were washed once with warm 1× PBS, and the media were replaced with antibiotic-free MEM-α + 10% FBS with CMG 14-12 supernatant at 1:20 vol/vol. The bacterial inocula were prepared from WT AH1263 and adjusted to a concentration of approximately 5×10^8^ CFU/mL in sterile 1× PBS. Cell monolayers were infected with S. aureus by adding 1.25×10^6^ CFU in 2.5 μL for a multiplicity of infection (MOI) of 25. PBS alone was added to control wells as a mock-infection. The plates were centrifuged at 1,000 rpm for 1 min to bring bacteria in contact with the cell monolayers. The plates were then incubated for 2 h at 37°C to allow for bacterial internalization. The media were then removed, and cells were washed in warm 1× PBS. Cells were incubated for an additional hour with fresh MEM-α supplemented with 100 μg/mL gentamicin to kill extracellular bacteria. Following gentamicin protection, one set of samples was immediately lysed in 0.1% Triton-X in 1× PBS to enumerate the internalized CFU. Cell supernatants were plated from the infected wells to ensure that gentamicin effectively killed the extracellular bacteria. A separate set of infected cells was transitioned to BMDM maintenance media and incubated at 37°C and 5% CO_2_ for 2 more days, after which time infected cells were either lysed for enumeration of the intracellular bacterial load or fixed and TRAP-stained. The TRAP-stained wells were also stained with DAPI to visualize nuclei, and they were quantified in the same manner as the osteoclastogenesis assays.

### Murine model of osteomyelitis.

All animal experiments were approved by the Vanderbilt University Institutional Animal Care and Use Committee. Mice underwent induction of posttraumatic osteomyelitis as previously described (43, 44, 53). Briefly, 7 to 8-week-old mice were anesthetized using 3 to 3.5% isoflurane for induction and 1 to 1.5% for maintenance. The mice received subcutaneous injections of sustained-release buprenorphine (0.5 to 1 mg/kg) prior to the procedure and to maintain analgesia for 48 h postinfection. After the induction of anesthesia, the left hindlimb and flank were prepared for surgery, and the diaphysis of the femur was exposed using sterile techniques. An approximately 1 mm defect was made in one side of the cortical bone via trephination with a 21-gauge needle. An inoculum containing either 1×10^5^ CFU or 1×10^6^ CFU of AH1263 in 2 μL of PBS was injected into the cortical defect using a micropipette. The muscle fascia and skin were then closed with sutures, and the mice were allowed to recover from anesthesia. Mice were monitored and weighed daily until the experimental endpoint. Mice were monitored for humane endpoint criteria, which included an inability to eat or drink, immobility or lethargy, hunched posture, and weight loss of greater than 20% body weight occurring after 4 days postinfection. None of the mice in this study met the criteria for humane endpoint euthanasia. At the experimental endpoint, mice were euthanized. In the event that a femur was fractured during extraction, the sample was excluded from the imaging analyses.

### CFU enumeration.

At the desired endpoint, femurs, kidneys, and livers were sterilely dissected from euthanized mice that were subjected to osteomyelitis. Femurs or kidneys were singly placed in NAVY bead lysis tubes (Next Advance, Troy, NY) containing 500 μL of sterile 1× PBS. Because of their larger sizes, the livers were split into two bead lysis tubes prior to homogenization. The tissues were then homogenized at 4°C using a Bullet Blender at the highest setting for 3 intervals of 5 min each. The homogenates were serially diluted in sterile 1× PBS and plated on TSA plates. The plates were incubated overnight at 30°C, and the CFU were counted the next day. The limit of detection for this workflow was 1.69 and 1.99 Log_10_ transformed CFU for the femurs/kidneys and livers, respectively.

### Microcomputed tomography (μCT) of cortical and trabecular bone in femurs.

Femurs were dissected from mice at day 14 postinfection, fixed in 10% neutral buffered formalin for 48 h, and then moved to 70% EtOH before storage at 4°C. Fixed femurs were scanned using a μCT50 (Scanco Medical, Switzerland) instrument and analyzed with μCT Tomography V6.3-4 software (Scanco USA, Inc.). The scans were acquired at a 10.0 μm voxel size at 70 kV, 200 μA, and an integration time of 350 ms in a 10.24 mm view to result in 1,088 image slices. To accommodate the analysis of trabecular and cortical bone, a region of interest was selected on each femur to encompass the trabecular bone of the distal epiphysis, as well as the entire diaphysis, so that the cortical defect into which bacteria were inoculated could be visualized. The proximal epiphysis was excluded. Cortical bone destruction, callus formation, and trabecular bone volume were determined by contouring the indicated regions of interest as previously described (43, 44, 72). Briefly, to analyze cortical bone loss and callus formation, 818 slices were contoured, centering around the midpoint of the defect. For trabecular bone analysis, measurements to the distal trabecular bone were standardized to begin 30 slices above the growth plate. 101 slices were analyzed in total.

### Bone histology and histomorphometric analysis of osteoclasts in trabecular bone.

After imaging by μCT, fixed femurs were decalcified for 4 days in 20% EDTA (pH 7.4) at 4°C, with a solution change occurring on day 2. Decalcified femurs were processed into paraffin, embedded, and then sectioned at a thickness of 4 μm through the infectious nidus and bone marrow cavity using a Leica RM2255 microtome. The sectioned femurs were stained for TRAP with hematoxylin counterstain. Slides containing TRAP-stained histologic sections were then imaged at 10× on a Biotek Cytation 5 Image Reader (Agilent, Winooski, VT). Histomorphometry was performed on the imaged slides using BIOQUANT OSTEO software (BIOQUANT, Nashville TN). The analysis was performed on a region of interest of trabeculae proximal to the growth plate in the distal femur. Guided by ASBMR standards, the osteoclast surface and bone perimeter were calculated to quantify the percent osteoclast surface/bone surface (52).

### Statistical analysis.

For all experiments, the data were checked for normality prior to statistical analysis via either the D’Agostino-Pearson test or the Shapiro-Wilk test. In comparisons of two groups, including the comparisons of the μCT data and the CFU burdens in femurs and organs, a parametric *t* test or a nonparametric Mann-Whitney U test was used to make comparisons between groups, depending on the normality of the data distribution. To assess the relative contributions of genotype and limb type to trabecular bone volume, a repeated measures two-way ANOVA with Sidak’s test of multiple comparisons was used. To assess the effects of treatment and genotype on the osteoclast counts, a two-way ANOVA was used with a *post hoc* Dunnett’s test of multiple comparisons or Sidak’s test, depending on the comparison made. To assess the effect of genotype on the osteoclast count normalized to the vehicle, a one-way ANOVA was used with Dunnett’s test of multiple comparisons to make *post hoc* comparisons between genotypes. To assess the effects of genotype and RANKL-stimulation on the release of cytokines, a two-way ANOVA was used with Dunnett’s test of multiple comparisons to compare each genotype to the WT for each RANKL stimulation.
