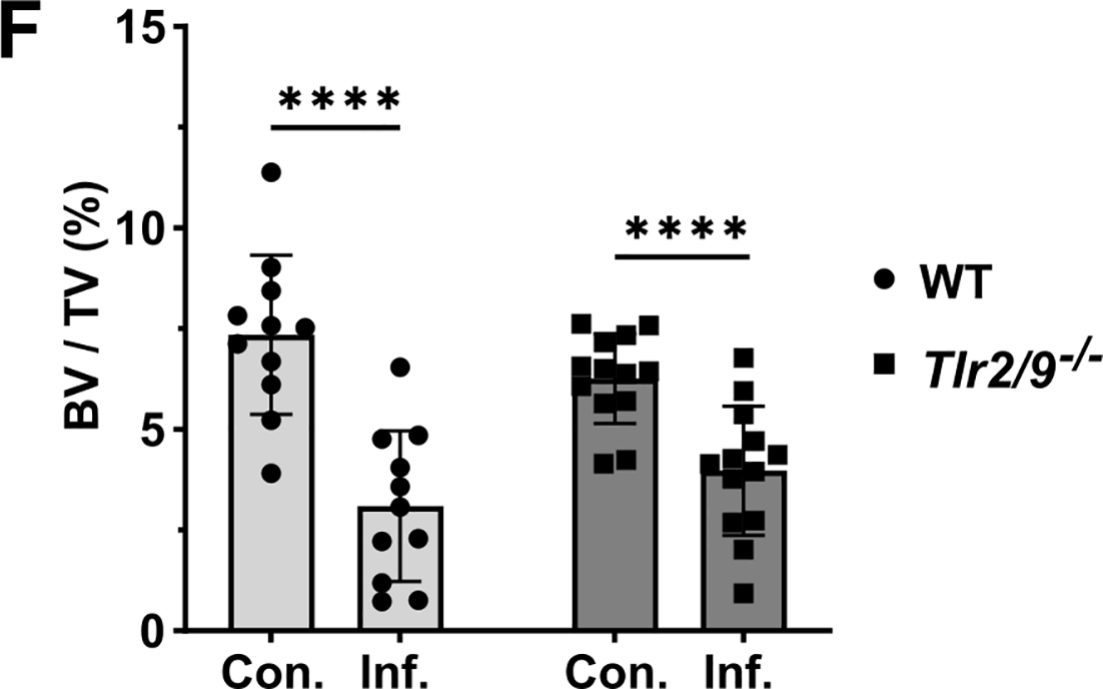# Erratum for Petronglo et al., “Context-Dependent Roles for Toll-Like Receptors 2 and 9 in the Pathogenesis of *Staphylococcus aureus* Osteomyelitis”

**DOI:** 10.1128/iai.00633-25

**Published:** 2026-04-15

**Authors:** Jenna R. Petronglo, Nicole E. Putnam, Caleb A. Ford, Virginia Cruz-Victorio, Jacob M. Curry, Casey E. Butrico, Laura E. Fulbright, Joshua R. Johnson, Sun H. Peck, Sana R. Fatah, James E. Cassat

## ERRATUM

Vol. 90, no. 11, e00417-22, 2022, https://doi.org/10.1128/iai.00417-22. Figure 4F should appear as shown in this correction. Some of the data points in the rightmost two bars of the graph were hidden behind the shaded bars. These formatting issues did not affect the data points, statistical analyses, or conclusions of the original manuscript.

Figure 1C: “2^ΔΔCq^” should read as “2^−ΔΔCq^” in the *y*-axis label for each of the four graphs.

**Fig 4:**