# Supplementary material for: Context-Dependent Roles for Toll-Like Receptors 2 and 9 in the Pathogenesis of Staphylococcus aureus Osteomyelitis
Source: Infect Immun. 2022 Oct 13;90(11):e00417-22. doi: 10.1128/iai.00417-22 (PMC9670883; doi:10.1128/iai.00417-22)
Supplement: Supplemental file 1 — Fig. S1 to S4 and Tables S1 to S5. Download iai.00417-22-s0001.pdf, PDF file, 0.5 MB [file iai.00417-22-s0001.pdf]

Supplementary Figures and Tables

Context-dependent roles for Toll-like receptors 2 and 9 in the pathogenesis of *Staphylococcus aureus* osteomyelitis

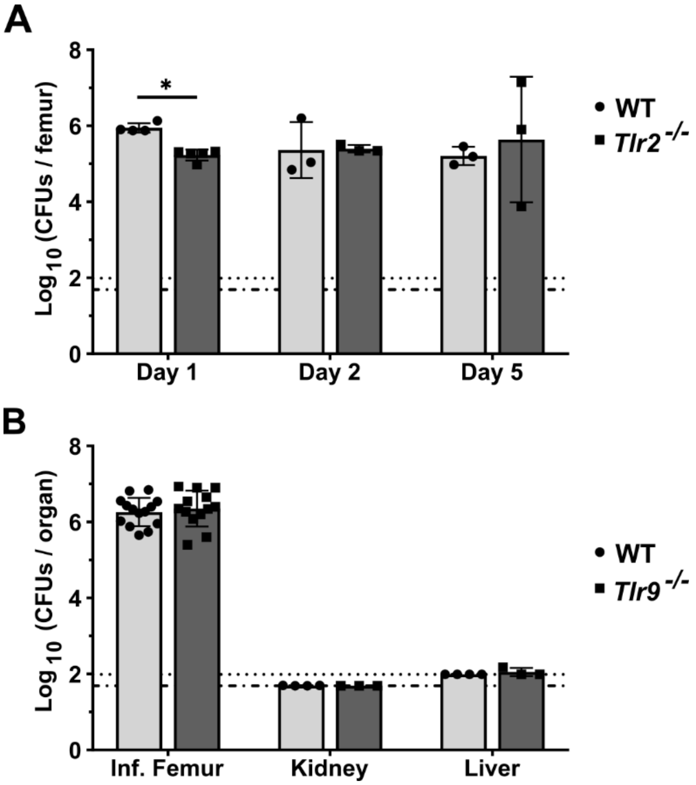

Supplemental Figure 1. Mice deficient in TLR2 or TLR9 do not incur increased bacterial burdens during osteomyelitis. A-B) WT and *Tlr2*$^{-/-}$ mice **(A)** or WT and *Tlr9*$^{-/-}$ mice **(B)** were subjected to osteomyelitis by intraosseous injection of 10^5 (**A**) or 10^6 **(B)** CFUs of WT *S. aureus*. Infected femurs, kidneys, and livers were extracted on the indicated day post-infection **(A)** or on day 14 post-infection **(B)**. Organ homogenates were plated for CFU enumeration. Error bars denote SD. Dotted lines indicate Log transformed limits of detection. **A)** Femurs were harvested at day 1 (WT n = 4, *Tlr2*$^{-/-}$ n = 5), day 2 (WT n = 3, *Tlr2*$^{-/-}$ n = 3), or day 5 (WT n = 3, *Tlr2*$^{-/-}$ n = 3). CFU burdens were compared between genotypes by Mann-Whitney U test. *p < 0.05. If not denoted with asterisks, statistical difference between genotypes was not significant. **B)** CFU burdens were compared by t-test (femur) or Mann-Whitney U test (liver, kidney). If not denoted with asterisks, statistical

difference between genotypes was not significant. Results compiled from 3 independent experiments. For femurs, n = 15 for WT and for n = 14 for *Tlr9*$^{-/-}$. For liver and kidneys, n = 4 for WT and n = 3 for *Tlr9*$^{-/-}$.

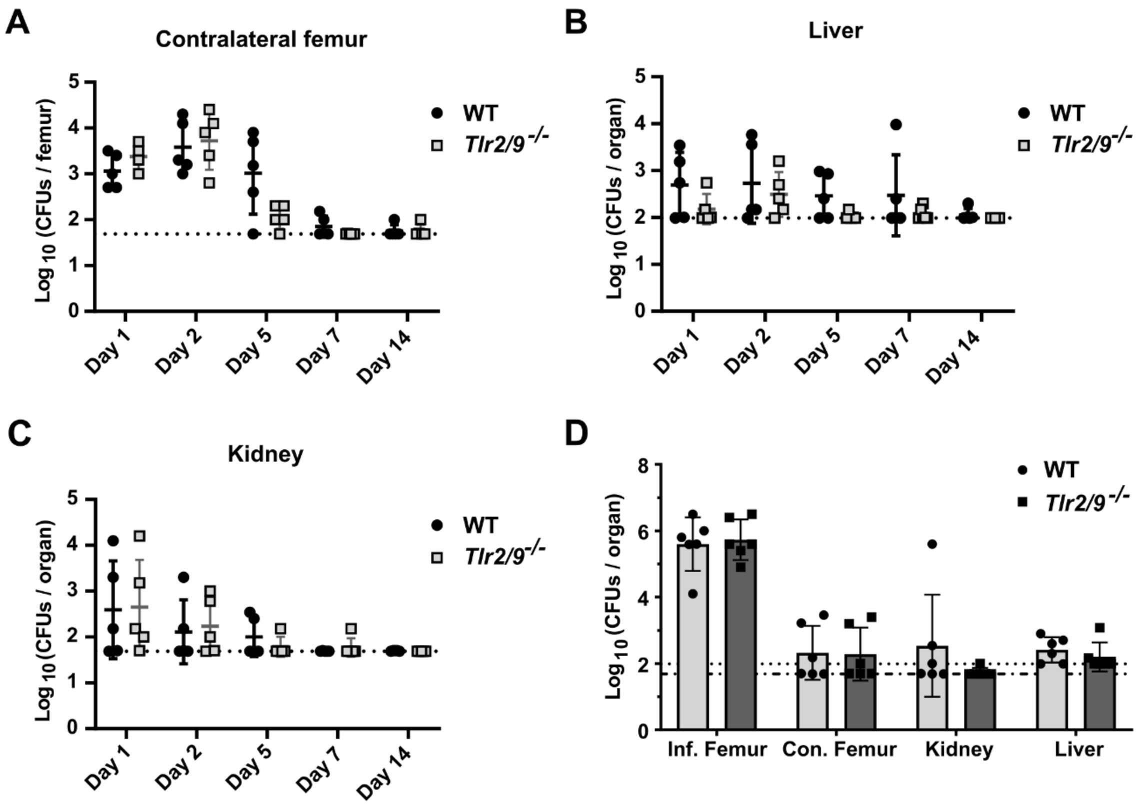
A
Contralateral femur
Log10 (CFUs / femur)
WT
Tlr2/9-/-
Day 1
Day 2
Day 5
Day 7
Day 14
B
Liver
Log10 (CFUs / organ)
C
Kidney
D
Inf. Femur
Con. Femur
Kidney
Liver

Supplemental Figure 2. Mice deficient in TLR2 and TLR9 do not incur increased bacterial burdens over multiple infection time points. A-C) WT and *Tlr2/9*$^{-/-}$ mice were subjected to osteomyelitis by intraosseous injection of 10^6 CFUs of *S. aureus*. Mice were euthanized on the indicated day post-infection and organs were collected for CFU enumeration. CFU burdens from each organ were compared between genotypes for each time point using a t-test or Mann-Whitney U test, depending on normality of the data. *$p < 0.05$. If not denoted with asterisks, statistical difference between genotypes was not significant. Error bars denote SD. Dotted lines represent the Log_{10} transformed limits of detection. For days 1, 2, 5, and 14, n = 5 per group. For day 7, n = 6 per group. **D)** WT and *Tlr2/9*$^{-/-}$ mice were subjected to osteomyelitis by intraosseous injection of 10^5 CFUs of WT *S. aureus*. Mice were euthanized on day 7 post-infection. Organs were extracted and homogenized for CFU enumeration. CFU burdens were compared between genotypes using a Mann-Whitney U test. No significant differences were detected. n = 5 per group. Error is plotted as ± SD. Dotted lines indicate Log_{10} transformed limits of detection.

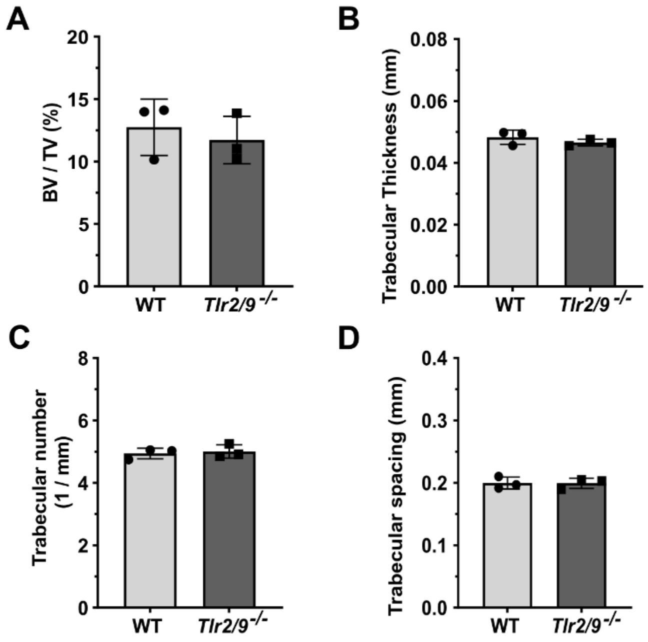

Supplemental Figure 3. WT and *Tlr2/9*$^{-/-}$ mice have comparable trabecular bone measurements at baseline. 8-9-week-old female mice were euthanized, and femurs were extracted, fixed, and imaged by µCT. Parameters of trabecular bone including %-BV/TV (**A**), trabecular thickness (**B**), number (**C**), and spacing (**D**) were measured. Comparisons were made between genotypes by Mann-Whitney U test. No significant differences were detected. n = 3 per group. Error bars denote SD.

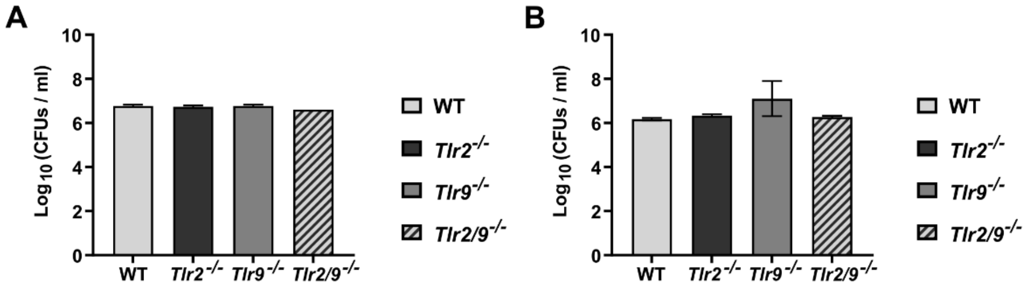

Supplemental Figure 4. Bacterial load is not affected by loss of TLR2 and/or 9. A-B) BMDMs of the indicated genotype were primed with RANKL + CMG 14-12 supernatant for 2 days. RANKL was withdrawn and cells were infected with WT *S. aureus* at a MOI of 25. CFUs were enumerated from cell lysates at the indicated time point. Dotted lines indicate the limit of detection. For all, n = 3 technical replicates per group. **A)** Log_{10} CFUs were analyzed by nonparametric one-way ANOVA. A Kruskal-Wallis post-hoc test was used to compare CFUs between knockout and WT cells and no significant differences were detected. **B)** After 2 days of infection, cells were lysed, serially diluted, and plated to measure CFUs. Log_{10} CFU burdens were analyzed by non-parametric one-way ANOVA. No significant differences were detected.

Supplemental Table 1. BMDMs were cultured in media containing 35 ng/ml RANKL + CMG 14-12 supernatant for 2 days. Cells were then changed to RANKL-free media and stimulated with 7% vol/vol of supernatant from the ∆*psmα1-4* ∆*spa* strain. Cytokine responses were measured after 12 h of stimulation. Average cytokine in pg/ml is shown ± standard deviation (SD).

*Under LOD measurement used in mean and SD calculation or only measured value reported

#One or more measurements omitted from mean and SD calculation because the coefficient of variation for duplicate samples was greater than 30% or because the measured value exceeded the detection maximum.

Supplemental Table 1: RANKL → No RANKL					
**Cytokine**	**WT vehicle**	**WT Δ*psmα1-4* Δ*spa***	***Tlr2*$^{-/-}$ Δ*psmα1-4* Δ*spa***	***Tlr9*$^{-/-}$ Δ*psmα1-4* Δ*spa***	***Tlr2/9*$^{-/-}$ Δ*psmα1-4* Δ*spa***
**G-CSF**	3.24 ± 0.29	2235.67 ± 258.11	8.85 ± 6.74	3059.67 ± 150.07	3.06 ± 0.47
**GM-CSF**	30.21 ± 1.76	29.58 ± 5.60	24.17 ± 7.50	31.93 ± 3.19	26.05 ± 1.84
**INFγ**	2.27 ± 0.27	3.29 ± 0.32	1.86 ± 0.75	4.36 ± 0.75	1.65 ± 0.10
**IL-1α**	28.57 ± 2.78	63.17 ± 13.08	13.46 ± 7.48	79.29 ± 17.29	27.97 ± 8.19
**IL-1β**	2.60 ± 0.84	2.46 ± 0.58	1.81 ± 0.66	3.57 ± 0.52	*1.75 ± 0.75
**IL-2**	11.86 ± 0.47	9.37 ± 0.35	9.22 ± 1.43	12.12 ± 5.38	9.74 ± 2.37
**IL-4**	$^{\#}$0.75 ± 0.19	*0.70 ± 0.16	0.63 ± 0.12	0.79 ± 0.23	*0.55 ± .01
**IL-5**	7.03 ± 1.99	11.66 ± 2.13	5.94 ± 1.50	$^{\#}$13.50 ± 0.27	6.15 ± 1.86
**IL-6**	5.10 ± 1.31	129.47 ± 11.96	6.31 ± 3.50	140.89 ± 13.06	5.68 ± 2.15
**IL-7**	2.27 ± 0.65	2.16 ± 0.54	1.85 ± 0.46	2.76 ± 1.11	1.88 ± 0.55
**IL-9**	67.59 ± 5.83	69.10 ± 7.39	41.74 ± 11.86	72.54 ± 18.84	48.36 ± 10.83
**IL-10**	3.90 ± 0.91	221.52 ± 30.61	4.27 ± 1.35	213.15 ± 3.82	4.38 ± 0.36
**IL-12 (p40)**	2.11 ± 0.80	2.64 ± 0.45	1.83 ± 0.95	4.49 ± 1.23	2.09 ± 0.54
**IL-12 (p70)**	13.73 ± 2.92	11.52 ± 3.36	8.74 ± 1.27	14.70 ± 4.21	8.37 ± 3.97
**IL-13**	2.56 ± 0.16	3.70 ± 0.54	1.16 ± 0.14	3.65 ± 0.75	1.62 ± 0.72
**IL-15**	14.71 ± 3.41	19.96 ± 5.68	13.02 ± 5.11	21.72 ± 7.95	14.70 ± 1.86
**IL-17**	0.77 ± 0.11	15.63 ± 0.89	*0.60 ± 0.14	13.30 ± 0.50	*0.64 ± 0.21
**IP-10**	251.70 ± 49.17	428.80 ± 86.71	115.70 ± 10.57	345.77 ± 78.95	188.17 ± 32.92
**KC**	29.39 ± 1.50	1345.33 ± 208.46	50.10 ± 14.27	1397.33 ± 240.35	31.39 ± 2.11
**MCP-1**	194.88 ± 23.34	1257.00 ± 143.95	217.59 ± 18.15	1063.87 ± 122.33	319.46 ± 9.10
**MIP-1α**	32.39 ± 1.73	1718.00 ± 239.06	50.79 ± 11.99	1181.67 ± 19.22	44.94 ± 4.76
**MIP-1β**	51.12 ± 4.46	8136.67 ± 1852.34	88.19 ± 10.37	5285.00 ± 52.46	78.43 ± 5.50
**MIP-2**	92.75 ± 8.76	13168.00 ± 1174.41	636.84 ± 44.73	$^{\#}$13164.00	324.89 ± 47.91
**RANTES**	3.96 ± 0.54	13.32 ± 1.42	2.76 ± 0.60	13.12 ± 0.55	3.39 ± 0.41
**TNFα**	5.06 ± 0.81	27.59 ± 1.80	6.38 ± 1.38	28.40 ± 1.81	5.04 ± 0.43

Supplemental Table 2. BMDMs were cultured in media containing CMG 14-12 supernatant for 2 days. Cells were then stimulated with 7% vol/vol of supernatant from the ∆*psmα1-4* ∆*spa* strain. Cytokine responses were measured after 12 h of stimulation. Average cytokine in pg/ml is shown ± SD.

*Under LOD measurement used in mean and SD calculation or only measured value reported

#One or more measurements omitted from mean and SD calculation because the coefficient of variation for duplicate samples was greater than 30% or because the measured value exceeded the detection maximum

<X All measurements were under detection minimum

>X All measurements were over detection maximum

Supplemental Table 2: No RANKL → No RANKL					
**Cytokine**	**WT vehicle**	**WT Δ*psmα1-4* Δ*spa***	***Tlr2*$^{-/-}$ Δ*psmα1-4* Δ*spa***	***Tlr9*$^{-/-}$ Δ*psmα1-4* Δ*spa***	***Tlr2/9*$^{-/-}$ Δ*psmα1-4* Δ*spa***
**G-CSF**	*0.89 ± 0.30	2951.67 ± 284.82	*1.05 ± 0.41	3657.67 ± 515.04	0.93 ± 0.15
**GM-CSF**	12.70 ± 3.80	*13.38 ± 8.20	*7.56 ± 3.33	25.38 ± 5.67	11.45 ± 1.01
**INFγ**	*0.83 ± 0.34	2.18 ± 0.52	*0.76 ± 0.23	3.39 ± 0.61	*0.63 ± 0.16
**IL-1α**	11.31 ± 4.94	74.09 ± 9.49	*11.14± 6.01	96.79 ± 2.53	13.74 ± 6.08
**IL-1β**	*1.197 ± 0.51	2.16 ± 0.93	<0.90	3.58 ± 2.24	<0.90
**IL-2**	3.83 ± 0.80	4.85 ± 1.34	#7.93 ± 1.05	9.03 ± 2.55	5.81 ± 1.71
**IL-4**	<0.55	<0.55	<0.55	<0.55	<0.55
**IL-5**	2.02 ± 0.83	3.49 ± 1.13	1.37 ± 0.32	4.54 ± 0.68	2.01 ± 0.13
**IL-6**	0.85 ±	265.87 ± 44.93	1.32± 0.92	512.12 ± 177.25	1.46 ± 0.37
**IL-7**	<0.77	<0.77	<0.77	1.28 ± 0.12	*0.78 ± 0.02
**IL-9**	15.15 ± 6.29	59.97 ± 9.00	19.78 ± 12.84	72.12 ± 7.96	15.17 ± 3.20
**IL-10**	1.61 ± 0.12	304.30 ± 30.61	*1.73 ± 0.88	313.69 ± 19.98	1.41 ± 0.42
**IL-12 (p40)**	*1.73 ± 1.26	*2.22 ± 1.56	*1.14	3.07 ± 0.26	2.44 ± 0.65
**IL-12 (p70)**	*1.86 ± 0.88	*3.50 ± 2.39	4.02 ± 0.55	6.30 ± 2.46	3.64 ± 0.77
**IL-13**	2.93 ± 0.89	4.36 ± 0.95	#1.48 ± 0.10	7.16 ± 1.46	2.10 ± 0.96
**IL-15**	*4.02 ± 3.66	*3.41 ± 1.59	3.26 ± 1.97	8.28 ± 3.32	3.36 ± 2.33
**IL-17**	2.74 ± 0.67	26.00 ± 1.80	#1.83 ± 0.64	28.04 ± 1.92	1.41 ± 0.13
**IP-10**	632.80 ± 170.51	483.16 ± 122.59	#273.16 ± 21.58	757.18 ± 162.12	477.56 ± 253.45
**KC**	67.46 ± 16.55	3020.67 ± 578.69	87.73 ± 17.13	4079.00 ± 465.54	66.83 ± 13.38
**MCP-1**	721.07 ± 30.04	2511.00 ± 398.65	#620.58 ± 17.79	4016.00 ± 1535.26	878.09 ± 71.08
**MIP-1α**	226.94 ± 18.88	#9878.00	#200.56 ± 1.41	>15053	168.96 ± 13.96
**MIP-1β**	728.12 ± 85.37	>9967	#611.94 ± 26.57	>9967	463.52 ± 36.68
**MIP-2**	462.50 ± 72.45	>14319	1993.67 ± 295.11	>14319	1142.00 ±108.57
**RANTES**	4.47 ± 1.24	56.32 ± 7.26	2.81 ± 1.44	66.56 ± 10.24	4.12 ± 1.09
**TNFα**	5.68 ± 0.92	101.80 ± 12.72	7.34 ± 1.61	32.12 ± 21.75	5.34 ± 0.46

Supplemental Table 3. BMDMs were cultured in media containing 35 ng/ml RANKL + CMG 14-12 supernatant for 2 days. RANKL was continued and cells were stimulated with 7% vol/vol of supernatant from the ∆*psmα1-4* ∆*spa* strain. Cytokine responses were measured after 12 h of stimulation. Average cytokine in pg/ml is shown ± SD.

*Under LOD measurement used in mean and SD calculation or only measured value reported

#One measurement omitted from mean and SD calculation because the coefficient of variation for duplicate samples was greater than 30%.

Supplemental Table 3: RANKL → RANKL					
**Cytokine**	**WT vehicle**	**WT *Δpsmα1-4 Δspa***	***Tlr2$^{-/-}$* *Δpsmα1-4 Δspa***	***Tlr9$^{-/-}$* *Δpsmα1-4 Δspa***	***Tlr2/9$^{-/-}$* *Δpsmα1-4 Δspa***
**G-CSF**	#2.62 ± 0.00	1556.00 ± 448.64	3.73 ± 0.82	1771.00 ± 251.81	4.92 ± 1.20
**GM-CSF**	29.17 ± 8.09	*22.27 ± 16.62	32.70 ± 1.47	28.34 ± 4.49	29.45 ± 1.93
**INFγ**	1.67 ± 0.25	2.23 ± 0.88	2.61 ± 0.39	2.74 ± 0.18	2.31 ± 0.39
**IL-1α**	29.51 ± 15.56	67.61 ± 22.26	48.67 ± 10.47	73.12 ± 6.90	39.94 ± 8.50
**IL-1β**	1.83 ± 1.13	*2.30 ± 1.28	2.59 ± 0.28	3.43 ± 0.66	1.97 ± 0.59
**IL-2**	9.70 ± 0.68	14.22 ± 1.11	18.49 ± 2.98	14.09 ± 2.63	15.77 ± 3.80
**IL-4**	*0.60 ± 0.09	*0.71 ± 0.09	0.86 ± 0.13	0.66 ± 0.00	0.63 ± 0.04
**IL-5**	6.44 ± 2.08	8.25 ± 1.60	7.32 ± 1.73	7.11 ± 1.09	6.97 ± 1.35
**IL-6**	#4.86 ± 0.47	94.94 ± 30.76	7.23 ± 2.43	106.23 ± 12.77	8.94 ± 1.47
**IL-7**	2.34 ± 1.55	*1.58 ± 0.74	2.76 ± 0.54	2.37 ± 0.13	2.44 ± 0.35
**IL-9**	55.79 ± 8.09	66.22 ± 10.71	79.82 ± 7.57	77.21 ± 6.63	73.25 ± 10.50
**IL-10**	4.69 ± 1.65	253.46 ± 44.59	11.26 ± 0.76	249.53 ± 11.41	9.29 ± 2.01
**IL-12 (p40)**	*2.12 ± 1.25	*1.90 ± 1.04	3.61 ± 0.70	3.57 ± 0.29	2.19 ± 0.45
**IL-12 (p70)**	#12.21 ± 0.59	10.83 ± 3.99	14.84 ± 0.96	15.94 ± 2.48	16.22 ± 3.13
**IL-13**	3.08 ± 0.46	3.10 ± 1.48	2.17 ± 0.67	3.36 ± 0.71	2.09 ± 0.31
**IL-15**	14.62 ± 11.88	*13.39 ± 10.18	19.18 ± 4.09	21.32 ± 1.22	23.27 ± 6.51
**IL-17**	*0.65 ± 0.13	12.23 ± 2.68	0.77 ± 0.13	9.39 ± 1.06	0.81 ± 0.07
**IP-10**	455.73 ± 110.01	425.95 ± 250.95	188.92 ± 67.60	329.83 ± 89.40	205.14 ± 54.59
**KC**	#38.86 ± 1.08	984.60 ± 239.27	52.59 ± 2.14	1051.55 ± 119.45	45.36 ± 4.29
**MCP-1**	174.03 ± 27.57	932.05 ± 255.42	161.24 ± 5.99	776.28 ± 42.69	247.82 ± 6.68
**MIP-1α**	30.15 ± 3.95	925.49 ± 188.22	38.05 ± 3.92	650.46 ± 35.97	37.42 ± 0.23
**MIP-1β**	45.99 ± 8.48	3931.33 ± 1472.54	65.75 ± 4.46	2186.00 ± 259.28	65.24 ± 1.70
**MIP-2**	91.93 ± 25.85	10484.67 ± 2100.01	746.96 ± 30.80	13295.33 ± 1008.15	503.89 ± 46.82
**RANTES**	4.17 ± 0.48	11.17 ± 2.96	4.03 ± 0.28	11.34 ± 1.35	4.36 ± 0.08
**TNFα**	6.95 ± 0.91	25.08 ± 4.52	9.67 ± 1.16	27.46 ± 1.00	9.34 ± 0.35

Supplemental Table 4. BMDMs were cultured in media with CMG 14-12 supernatant for 2 days. Cells were then moved to media containing 35 ng/ml RANKL + CMG 14-12 supernatant and stimulated with 7% vol/vol of supernatant from the ∆*psmα1-4* ∆*spa* strain. Cytokine responses were measured after 12 h of stimulation. Average cytokine in pg/ml is shown ± SD.

*Under LOD measurement used in mean and SD calculation or only measured value reported

#One or more measurements omitted from mean and SD calculation because the coefficient of variation for duplicate samples was greater than 30% or because the measured value exceeded the detection maximum.

>X All measurements were over detection maximum

Supplemental Table 4: No RANKL → RANKL					
**Cytokine**	**WT vehicle**	**WT Δ*psmα1-4* Δ*spa***	***Tlr2$^{-/-}$*** **Δ*psmα1-4* Δ*spa***	***Tlr9$^{-/-}$*** **Δ*psmα1-4* Δ*spa***	***Tlr2/9$^{-/-}$*** **Δ*psmα1-4* Δ*spa***
**G-CSF**	*0.86 ± 0.32	2786.00 ± 176.60	*1.10 ± 0.45	2507.33 ± 156.30	*0.99 ± 0.37
**GM-CSF**	13.42 ± 1.67	21.33 ± 2.46	8.32 ± 2.14	23.02 ± 2.96	*8.26 ± 3.77
**INFγ**	*0.68 ± 0.08	3.58 ± 1.02	0.86 ± 0.20	3.42 ± 1.18	*0.80 ± 0.28
**IL-1α**	12.68 ± 2.95	86.24 ± 12.75	16.46 ± 5.41	80.67 ± 6.67	14.64 ± 14.07
**IL-1β**	*1.11 ± 0.37	4.99 ± 0.35	*1.11 ± 0.37	4.22 ± 1.40	<0.90
**IL-2**	5.21 ± 1.19	10.30 ± 1.30	6.59 ± 2.86	6.86 ± 1.97	5.11 ± 3.39
**IL-4**	<0.55	<0.55	<0.55	<0.55	<0.55
**IL-5**	1.44 ± 0.42	3.64 ± 0.45	1.72 ± 0.24	3.04 ± 0.52	1.37 ± 0.12
**IL-6**	1.17 ± 0.58	364.28 ± 20.56	2.06 ± 0.74	481.89 ± 96.28	1.45 ± 0.58
**IL-7**	*0.88 ± 0.19	*0.84 ± 0.09	*0.83 ± 0.10	*0.86 ± 0.15	*0.88 ± 0.19
**IL-9**	17.10 ± 7.63	74.87 ± 5.26	18.73 ± 6.33	64.50 ± 5.38	17.16 ± 5.01
**IL-10**	2.90 ± 0.31	342.56 ± 40.25	3.24 ± 0.51	302.45 ± 13.55	1.94 ± 0.24
**IL-12 (p40)**	2.51 ± 0.75	4.24 ± 0.76	*1.01 ± 0.25	4.97 ± 0.68	1.79 ± 1.27
**IL-12 (p70)**	1.69 ± 0.40	#8.90 ± 4.09	*2.21 ± 0.86	4.54 ± 0.60	*2.41 ± 1.11
**IL-13**	4.70 ± 0.48	5.32 ± 0.62	1.58 ± 0.15	7.08 ± 1.81	1.92 ± 0.48
**IL-15**	*4.02 ± 3.66	8.48 ± 3.66	*3.22 ± 2.45	7.39 ± 4.17	*2.91 ± 1.38
**IL-17**	1.42 ± 0.10	27.74 ± 1.73	1.50 ± 0.45	26.85 ± 1.16	0.87 ± 0.17
**IP-10**	1477.00 ± 423.47	647.46 ± 92.92	300.88 ± 20.84	816.43 ± 195.73	398.81 ±76.14
**KC**	109.90 ± 21.33	3742.67 ± 468.76	90.86 ± 25.27	3921.67 ± 290.82	59.02 ± 13.01
**MCP-1**	733.01 ± 72.76	2742.33 ± 377.35	526.79 ± 31.64	3080.33 ± 276.73	752.70 ± 37.47
**MIP-1α**	167.32 ± 15.79	#14596	164.44 ± 31.64	#11801.50 ± 594.68	136.90 ± 7.27
**MIP-1β**	461.63 ± 46.86	>9967	441.94 ± 14.42	>9967	335.25 ± 29.34
**MIP-2**	637.85 ± 46.31	>14319	2116.33 ± 704.98	>14319	1144.40 ± 181.17
**RANTES**	6.21 ± 1.69	80.11 ± 9.63	3.38 ± 0.78	84.47 ±0.59	3.65 ± 1.25
**TNFα**	5.03 ± 0.69	127.58 ± 12.27	6.35 ± 2.39	122.10 ± 3.70	4.55 ± 0.95

Supplemental Table 5. Sequences for primers used in RT-qPCR.

Gene	Protein Target	Fwd	Rv	Source
Actb	β-Actin	5'-GCAAGTGCTTCTAGGCGGAC-3'	5"-AAGAAAGGGTGTAAAACGCAGC-3'	Citation 1.
Ctsk	Cathepsin-K	5'-GAAGAAGACTCACCAGAAGCAG-3'	5'-TCCAGGTTATGGGCAGAGATT-3'	Citations 2, 3-5. Primer Bank 31982433a1
Nfatc1	NFATc1	5'-CCCGTCACATTCTGGTCCAT-3'	5'-CAAGTAACCGTGTAGCTGCACAA-3'	Citation 6.
Oscar	OSCAR	5'-TGGCGGTTTGCACTCTTCA-3'	5'-GATCCGTTACCAGCAGTTCCAGA-3'	Citation 7.
Tnfrsf11a	RANK Receptor	5'-GGACGGTGTTGCAGCAGAT-3'	5'-GCAGTCTGAGTTCCAGTGGTA-3'	Citations 3-5. Primer Bank 31981958a1

References for Supplemental Table 5

1. An, D., Kim, K. & Lu, W. Defective Entry into Mitosis 1 (Dim1) Negatively Regulates Osteoclastogenesis by Inhibiting the Expression of Nuclear Factor of Activated T-cells, Cytoplasmic, Calcineurin-dependent 1 (NFATc1). *Journal of Biological Chemistry* **289**, 24366–24373 (2014).

2. Zhang, J. *et al.* Exercise-induced irisin in bone and systemic irisin administration reveal new regulatory mechanisms of bone metabolism. *Bone Res* **5**, (2017).

3. Spandidos, A., Wang, X., Wang, H. & Seed, B. PrimerBank: a resource of human and mouse PCR primer pairs for gene expression detection and quantification. *Nucleic Acids Res* **38**, D792–D799 (2010).

4. Spandidos, A. *et al.* A comprehensive collection of experimentally validated primers for Polymerase Chain Reaction quantitation of murine transcript abundance. *BMC Genomics* **9**, 1–17 (2008).

5. Wang, X. & Seed, B. A PCR primer bank for quantitative gene expression analysis. *Nucleic Acids Res* **31**, e154 (2003).

6. Luo, J. *et al.* LGR4 is a receptor for RANKL and negatively regulates osteoclast differentiation and bone resorption. *Nat Med* **22**, 539–546 (2016).

7. Cui, W. *et al.* CD200 and its receptor, CD200R, modulate bone mass via the differentiation of osteoclasts. *Proc Natl Acad Sci U S A* **104**, 14436 (2007).
